# Automated Composition Assessment of Natural Extracts: Untargeted Mass Spectrometry-Based Metabolite Profiling Integrating Semiquantitative Detection

**DOI:** 10.1021/acs.jafc.3c03099

**Published:** 2023-11-10

**Authors:** Adriano Rutz, Jean-Luc Wolfender

**Affiliations:** † School of Pharmaceutical Sciences, University of Geneva, 1211 Geneva, Switzerland; ‡ Institute of Pharmaceutical Sciences of Western Switzerland, University of Geneva, 1211 Geneva, Switzerland; § Institute of Molecular Systems Biology, ETH Zürich, 8093 Zürich, Switzerland

**Keywords:** metabolite profiling, Charged Aerosol Detection, automated composition assessment, liquid chromatography–mass spectrometry, natural extract

## Abstract

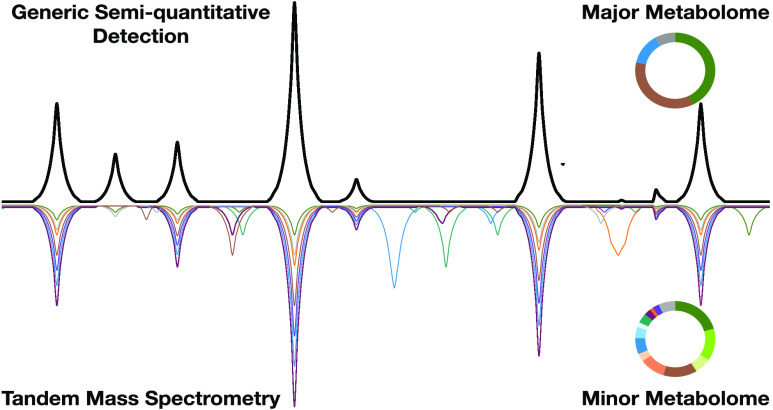

Recent developments in mass spectrometry-based metabolite profiling allow unprecedented qualitative coverage of complex biological extract composition. However, the electrospray ionization used in metabolite profiling generates multiple artifactual signals for a single analyte. This leads to thousands of signals per analysis without satisfactory means of filtering those corresponding to abundant constituents. Generic approaches are therefore needed for the qualitative and quantitative annotation of a broad range of relevant constituents. For this, we used an analytical platform combining liquid chromatography–mass spectrometry (LC–MS) with Charged Aerosol Detection (CAD). We established a generic metabolite profiling for the concomitant recording of qualitative MS data and semiquantitative CAD profiles. The MS features (recorded in high-resolution tandem MS) are grouped and annotated using state-of-the-art tools. To efficiently attribute features to their corresponding extracted and integrated CAD peaks, a custom signal pretreatment and peak-shape comparison workflow is built. This strategy allows us to automatically contextualize features at both major and minor metabolome levels, together with a detailed reporting of their annotation including relevant orthogonal information (taxonomy, retention time). Signals not attributed to CAD peaks are considered minor metabolites. Results are illustrated on an ethanolic extract of *Swertia chirayita* (Roxb.) H. Karst., a bitter plant of industrial interest, exhibiting the typical complexity of plant extracts as a proof of concept. This generic qualitative and quantitative approach paves the way to automatically assess the composition of single natural extracts of interest or broader collections, thus facilitating new ingredient registrations or natural-extracts-based drug discovery campaigns.

## Introduction

Recent developments in metabolomics and untargeted liquid chromatography–mass spectrometry (LC–MS) analyses allow unprecedented qualitative coverage.^1^ Automation and throughput of analyses have also been significantly improved.^2^ While these developments are crucial for better qualitative characterization of any sample, quantification remains limited to a few compounds.^3^ Accurate quantification is a tedious process that requires high-quality standards, making it unavailable on a large scale of compounds.^4^ Furthermore, although highly sensitive, LC–MS is not a universal detection technique.^5^ Given the sensitivity achieved by modern MS instruments, only a portion of the acquired signals is relevant for the compositional assessment.^6^ Indeed, out of all the signals considered in an LC–MS analysis, the majority are chemical adducts or fragments created during the ionization process and a single “analyte” can lead to dozens of features.^7^ This is true, especially for LC–ESI–MS, where some compounds can exhibit very large differences in their ionization behavior.^8^ This is a major drawback of current untargeted LC–MS approaches, as a large fraction of the annotated signals cannot be linked to any generic semiquantitative information. This is even worse for very rich matrices such as plant extracts, as a single analysis can suggest hundreds or thousands of features,^9^ without satisfactory means of filtering out the analytes that are significant, or that represent a substantial amount of the extracted mass. Despite many advantages in terms of sensitivity and metabolome coverage, untargeted LC–MS analyses are not yet adopted widely for natural extract compositional assessment due to the complexity of the generated data. It is, therefore, crucial to develop methodologies that provide semiquantitative information on a wide range of signals and that estimate annotation confidence.

### Confident Annotation of Multiple Features

The introduction of Feature-based Molecular Networking was a major step in deciphering the huge amount of data generated by MS-based metabolomics approaches.^10^ While spectral matching of features to spectral libraries had already existed for a long time,^11^ calculating the spectral similarity between features to organize and visualize them into spectral families has greatly facilitated the chemical study of complex samples.^12^ Further improvements have been made recently by Ion Identity Networking,^13^ and by linking positive ionization (PI) and negative ionization (NI) modes.^14^ Lately, Sirius^15^ has integrated several subtools into its ecosystem. The first, CANOPUS, predicts compound classes from mass spectra.^1^ Originally designed using the ClassyFire chemical taxonomy,^16^ it now calculates results based on the NPClassifier taxonomy, which is better suited for natural products (NPs).^17^ Since it is easier to predict a chemical class than a specific structure, the results of the chemical class annotation are more accurate than the structure annotation. However, until recently, there was no way to estimate the confidence in the annotation. For this, a Support Vector Machine approach was developed to estimate the confidence of metabolites absent from spectral libraries.^18^ Performance was then improved using deep neural networks.^19^ Another complementary taxonomy-based confidence scoring method was developed in-house.^20^ Together, these approaches can facilitate the filtering of highly trusted annotations. Such annotations, even if at a lower confidence level than internal libraries of standards,^21^ can be used as additional anchor points before further investigations.

### Generic Quantitation of Multiple Components

In MS, simultaneous quantitation on multiple (classes of) chemicals remains challenging. Some approaches have attempted to include optimally selected internal standards from various chemical families to extrapolate the ionization of these compound classes.^22^ These approaches remain study-specific and require the use of standards without completely overcoming the intrinsic limitations of compound ionization specificity. Therefore, the use of hyphenation with other types of detectors has emerged as a way to partly overcome these limitations and capture additional complementary information simultaneously. One way to improve the analysis was by combining LC–MS platforms with Photo Diode Array (PDA) detectors.^23^ This allowed for the recording of ultraviolet (UV) spectra and UV detectors are now routinely used coupled to LC. More recently, “universal” detectors like the Evaporative Light Scattering Detector (ELSD)^24^ and the Charged Aerosol Detector (CAD)^25^ have been introduced. These detectors can detect compounds that do not have chromophores and ideally provide a response that is not specific to a particular class of compounds. The CAD, introduced in 2005, has shown multiple advantages in comparison to ELSD.^26^ The CAD limit of detection ranges between 0.002^27^ and 0.5 μg/mL,^28^ depending on the studies, with most values reported around 0.05 μg/mL.^29,30^ In most articles, the limits of quantitation were approximately twice the value of the limit of detection. As the aim of this study is to remain as generic as possible, no compound-specific limit of detection will be determined.

Quantitative detectors have been used in gas chromatography (GC) for many years, in GC-Flame Ionization Detector (FID)-MS platforms.^31^ However, GC-FID is limited to the analysis of volatile substances. All three detectors (CAD, ELSD, and FID) mentioned above are one-dimensional detectors. They provide intensity measurements over time but no additional spectral dimensions, such as PDA or MS detection. As a result, achieving a high chromatographic resolution is necessary for these detectors, since the data cannot be deconvoluted using the third spectroscopic dimension. Therefore, ideally, all peaks should be separated well from each other. GC inherently offers higher chromatographic resolution than HPLC, making such approximations possible in GC-FID. In LC, achieving very high chromatographic resolution is still a challenge, but current UHPLC platforms have made significant progress in increasing peak capacity.^32^

### Combining Untargeted Annotation and Generic Quantitation

Taking advantage of the technological and methodological advancements mentioned above, this work aims to make sense of the abundant qualitative information from current untargeted LC–MS analysis pipelines and link it to universal semiquantitative information, as well as other complementary metadata, such as taxonomy. Such developments should facilitate an automated comprehensive assessment of the natural extract composition. Including the semiquantitative dimension early in the dereplication process can also facilitate the isolation of the compounds for further structural elucidation.^33^ The objective is to generate metabolite profiling data from natural extracts, categorizing them based on semiquantitative information that distinguishes between major and minor metabolome. Additionally, by grouping related features, the extensive MS data are condensed into meaningful information for each LC peak. While the hyphenation of both CAD and MS detectors was illustrated by Baker and Regg for botanical constituents determination in 2018, they mostly focused on the analytical aspects.^34^

Our approach aims to simplify the interpretation of the data. Our workflow and reporting mainly prepare the data for final inspection and further targeted confirmation, which is expected to be particularly useful for the registration of botanical ingredients and for the efficient isolation of new NPs. We particularly stress that our tool is only a guide to optimize further experiments required depending on the application (e.g., in applications necessitating accurate quantitation of given compounds to determine the instrumentation-specific limit of detection).

## Results and Discussion

The process leading to the qualitative and semiquantitative compositional assessment is briefly described below. As a minimal input, the workflow requires LC–MS/MS data, supplemented by another detector (here, CAD, as described in Data Acquisition). It then needs to be converted to an open format such as mzML, with all detectors encoded into the same file (Data Conversion). Multiple processing steps then follow and will be detailed in the next sections. First, signals are aligned as described in Signals’ Pretreatment. Difficult to integrate properly at first, CAD required additional processing, as described in CAD Processing. After several signal processing steps described below, a qualitative and semiquantitative report is generated, as well as visual means to interrogate the data. In parallel to the above-described semiquantitative processing, MS features are extracted, clustered, and annotated, as described in MS/MS Processing. Afterward, MS features are attributed to CAD peaks as described in the Link Between CAD and MS Data. This allows signals to be categorized as belonging to “major” (linked to CAD) or “minor” metabolites. Finally, to facilitate further investigations, different automated numerical and visual reports are generated as described in Data Visualization. Although the presented method can be adapted to any type of extract, all of the results are illustrated on an ethanolic extract of the aerial parts of *S. chirayita* (Roxb.) H. Karst. (Gentianaceae), presenting a typical compositional complexity.

### Data Acquisition

As previously mentioned, one strong requirement for efficient CAD integration is good chromatographic resolution. To achieve the best possible peak capacity while maintaining generic conditions, multiple trials were performed, with various representative plant extracts, columns, and gradient lengths (data not shown). For the example illustrated below, UHPLC runs of 126 min on a 150 mm column were performed. This corresponded to a peak capacity of 480 at 400 g/mol and 40 °C (for comparison, a run of 7 min on a 50 mm column corresponded to a peak capacity of 199, calculated with HPLC Calculator version 3.1).^35^ The details of the data acquisition method are available in Data Acquisition.

### Data Processing

**Figure 1:**
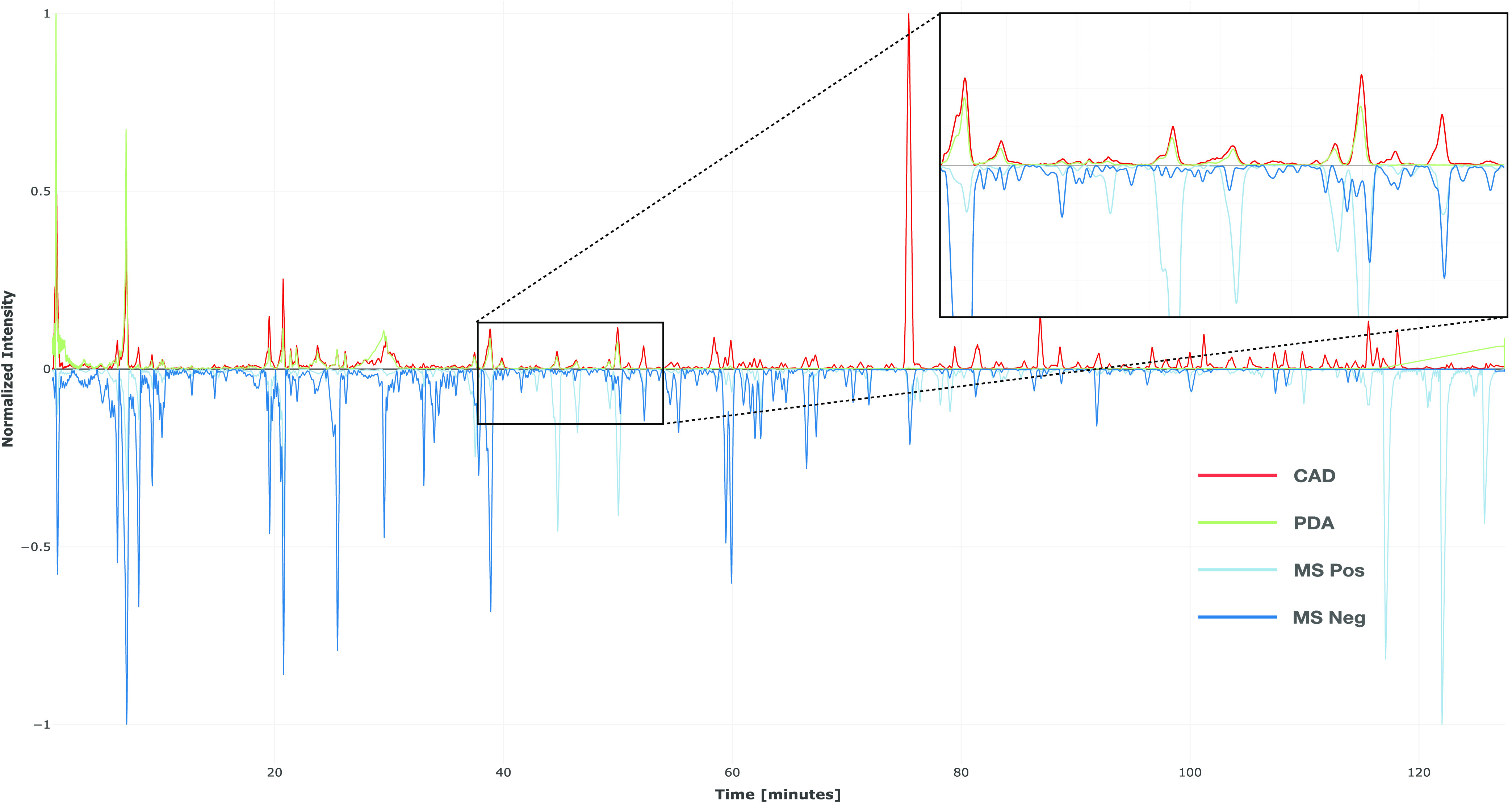
Aligned chromatograms after preprocessing. All signals were preprocessed to improve the downstream steps. CAD and PDA signals are at the top in red and green, respectively. MS signals (positive and negative ionization modes) are at the bottom in light and dark blue, respectively. A zoomed-in area showing the different intensities normalized to the observed signal is also illustrated.

Specific parsers were necessary for reading the CAD signal from the RAW file and translating it into the mzML file. The details are available in the section Data Conversion. Then, the signals of the different detectors were aligned. The instrumentation used consisted of a UHPLC connected to a PDA, a CAD, and an orbitrap MS. The nondestructive PDA was placed before an analytical split, and both CAD and MS detectors after. The MS signal was delayed by 0.090 and 0.055 min in comparison to the one of the PDA and the CAD, respectively. These values were used to align the signals as shown in Figure 1.

Figure 1 highlights the complementarity between each signal obtained. While the PDA intensity followed approximately the CAD intensity when a signal was present, some major molecules without a chromophore would have been ignored if PDA was used solely. The same complementarity could be observed between MS ionization modes (see light and dark blue traces). While each CAD major peak had its corresponding peak in MS, the opposite did not hold true, as illustrated by the positive ionization mode at the end of the chromatogram. In order to optimize coverage, it is strongly recommended to use both ionization modes, although the presented approach can also be applied to a single ionization mode.

#### Charged Aerosol Detector Data

As the CAD acquisition was set to capture small changes in the signal, it required some preprocessing steps before peak picking. These are briefly described hereafter and in more detail in the related Experimental Section. First, a Fourier transformation was performed to reduce noise as in ref (36). Moreover, the chromatographic resolution was improved using the derivative enhancement approach as described in ref (37). This resulted in a sharper signal, where peak detection could be performed with better results than those on the raw signal. To ensure the same processing between the different detectors, we also performed this on the PDA and BPI chromatograms.

As the CAD signal contained only one dimension, peaks were integrated by using simple algorithms. More elaborate techniques exist for multidimensional signals, such as the ones used in MS and PDA peak detection.^13,38−41^ A comparison of the peaks detected automatically before and after signal processing is shown in the Supporting Information. This allowed for a good CAD peak-shape comparison with MS features.

#### High-Resolution Tandem Mass Spectrometry Data

MS features were extracted using MZmine3 v.3.4.0.^42^ They were then annotated using the TIMA v.2.8.0,^20,43^ encompassing SIRIUS^15,18,44−46^ and ISDB annotations.^47^ Such an approach allows one to use the strengths of each tool and take advantage of their complementarity. In PI, this led to 3610 features, of which 3254 were annotated, and 1272 confidently. In NI, this led to 2312 features, of which 2003 were annotated, and 637 confidently. All relevant details can be found in the corresponding Experimental Sections.

#### Link Between CAD and MS Data

CAD and MS peak shapes had to be compared to select the features most likely to correspond to a given semiquantitative signal. As a first mandatory step, all points of CAD and MS peaks were inter- and extrapolated to the same frequency to be defined with approximately the same number of points. This is generally not needed, as peak shapes usually compared are coming from the same detector, resulting in a unique frequency of acquisition. Here, resampling was performed since the frequency of the CAD was approximately 30 times higher than the one of MS. Both chromatograms were resampled to a frequency of 2 Hz, which approximated the one of the MS1.

The second step consisted of normalizing the peak’s intensity together with a retention time normalization between the two peak’s minima (illustrated in green in Figure S1). The aim of this procedure was to improve peak-shape similarity between the compounds whose MS intensity strongly differs from that of the CAD trace. Without this step, the MS features with the highest intensity would by default have a more defined peak shape and, thus, be more likely to be correlated to the CAD signal. Only features with a similarity ≥0.8 were considered, with the possibility to adapt the threshold.

If an MS feature had a correlation ≥0.8, its corresponding annotation was considered as a “major” compound, else, it was categorized as a “minor” compound. This allowed for the distinction of major and minor features even if they were found below the same CAD peak. All features outside of a CAD peak were considered “minor” by default. The higher the peak capacity,^48^ the better the discrimination between minors and majors.

**Figure 2:**
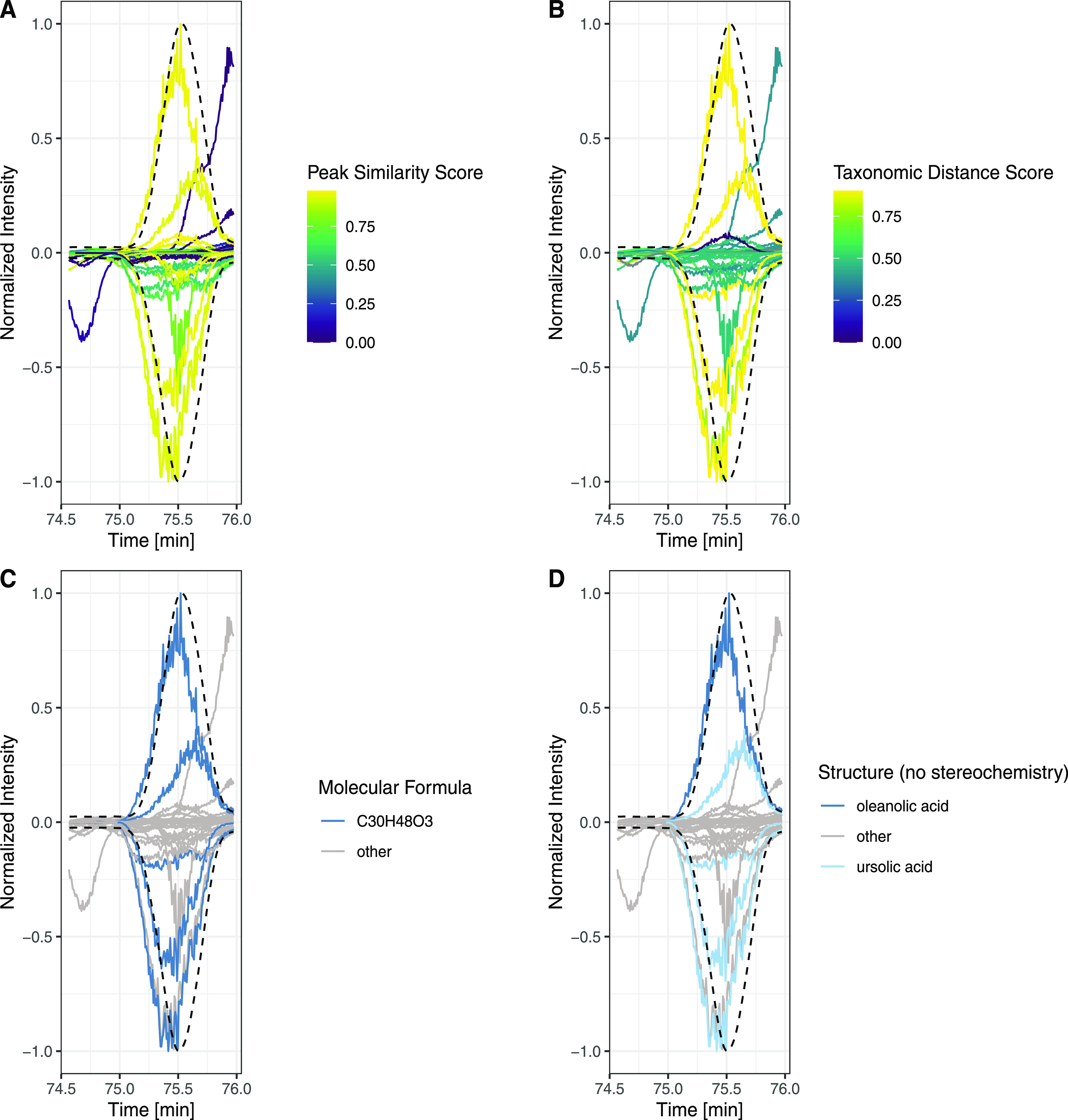
Link between MS features and CAD peak. Each panel is composed of positive and negative ionization modes (negative ionization modes are shown as negative values). The MS features are represented with plain lines and CAD peaks with dashed lines. In panel A, features are colored according to their peak-shape similarity with the CAD peak. In panel B, features are colored according to the taxonomicdistancescore part of the *taxonomically informed scoring*(20) (a value close to one indicates that the structure (without stereochemistry) has already been reported in a similar organism). In panel C, features are colored according to the MF of the best candidate. In panel D, features are colored according to the structure of the best candidate. Overall, this led to features that are more likely to be responsible for the CAD peak observed.

CAD peaks could envelop multiple or only a few (non)peak shape correlated features. An example of the most intense CAD peak of the *S. chirayita* extract (peak at 75 min in Figure 1) containing multiple MS features in both PI and NI modes is illustrated in Figure 2. Each panel is composed of PI and NI modes (NI mode is shown as negative values). The normalized extracted ion chromatograms of the MS features are represented with plain lines and CAD peaks with dashed lines; the colors of the lines depend on the factor illustrated.

First, in panel A, features were colored according to their peak-shape similarity to the CAD peak. Overall, more than 128 (67 PI and 61 NI) features were found below this CAD peak. Among the features sharing a good peak-shape similarity (≥0.8, in yellow), features with small intensity were found, together with a feature whose apex was slightly shifted in retention time. Features not sharing good peak-shape similarity were clearly distinguished. In panel B, features were colored according to the taxonomicdistancescore part of the *taxonomically informed scoring* (see Experimental Section).^20^ For example, a score of 0.9 indicates that the structure has already been reported in the same species as that studied (here, *S. chirayita*). Both chemical structures corresponding to oleanolic acid and ursolic acid without stereochemistry corresponded to structures already reported in *S. chirayita*.^49^ In panel C, features were colored according to the molecular formula (MF) of the best candidate. MFs with a good peak-shape similarity and a good taxonomic score are highlighted; other ones were grouped in the “other” category for visibility. This led to a unique MF, C_30_H_48_O_3_ (in dark blue), consistently annotated both in PI and NI, probably corresponding to different isomers, as suggested by the different traces observed. In panel D, features were colored according to the structure of the best candidate. This further refines panel C, showing that some of the features annotated with the same MF, C_30_H_48_O_3_, do not share the same structural annotation, oleanolic acid (dark blue) and ursolic acid (light blue). They were both triterpenoid derivatives, corresponding to the structures of oleanolic acid and ursolic acid, respectively. Together, the different relations between features and their respective CAD peak could help in gaining information while reducing the complexity of the data (from the initial 128 features, two major compounds could be highlighted). Informed filtering of the features considered as major metabolomes is detailed in the next section.

#### Major Compounds’ Filtering

Having all features informed as shown in Figure 2, a guided data reduction could be performed the same way on all peaks. This step was necessary to narrow the amount of information finally reported and analyzed. Basic statistics illustrating the number of features for PI, together with their link to their attributes, are given in Table 1.

**Table 1:** MS Features-CAD Peaks Statistics (Positive Ionization)

	no filter	peak-shape filter	peak-shape filter and taxon filter
picked features	3161	3161	3161
picked CAD peaks	62	62	62
features linked to CAD peak	1181	392	136
number of features per peak^a^	19.0	7.4	3.0
number of structures per peak^a^	16.6	6.6	2.6
number of MFs per peak^a^	16.6	6.6	2.6
number of chemical classes per peak^a^	13.4	5.9	2.5

a On average.

As the presented workflow aimed at linking MS features to CAD peaks to categorize them as “major” or “minor” metabolites, the fact that the features are under a CAD envelope was not sufficient. Filtering strategies were therefore developed to narrow down the number of features considered as “major.” The first step consisted of applying the peak-shape similarity as a filtering criterion as illustrated in Figure 2 (panel A).

As this filtering process was effective but not sufficient (over six structures/formulas per peak were assigned), an additional filter was implemented to further narrow down the number of features considered major. This filter consisted of favoring compounds already known in the genus of the studied extract. If the compound was already isolated from the same biological organism, then the probability of it being a major compound was considered higher. If a compound was found in the studied species, then all other “major” candidate features under the same CAD peak were attributed to the “minor” category. If multiple compounds were found at the species level (taxonomic distance score = 0.9), all of them were kept, as there was no rationale to choose one over another. The same rule was then applied to the genus level (taxonomic distance score of 0.8), giving priority to the compounds found at the species level. This led to fewer features considered “major”, with around two features remaining per CAD peak, as described in Table 1.

The corresponding table for NI mode is available in Appendix (Table S1). Roughly, 50 MS features were detected for 1 CAD peak. Overall, more features were detected in PI, compared to NI (3161 vs 2272). Not only the total features were higher in PI, but also the number of features per CAD peak. This is in line with previous conclusions, while PI mode is usually preferred as it is expected to be more generic, the ionization efficiency (ratio to noise) is better in NI mode.^50^

After the complete filtering process, on average, each CAD peak appeared to have an almost unambiguously attributed structure. The number of features and structures that could be attributed to a CAD peak was lowered by almost an order of magnitude (3.0 and 2.6 against 19.0 and 16.6 initially). In fact, even if there are annotated features under a CAD peak envelope before filtering, after filtering, some of the CAD peaks could not be linked any more to any confidently annotated and peak-shape correlated feature. Finally, only CAD peaks with linked features were therefore taken into account in the calculations.

**Figure 3:**
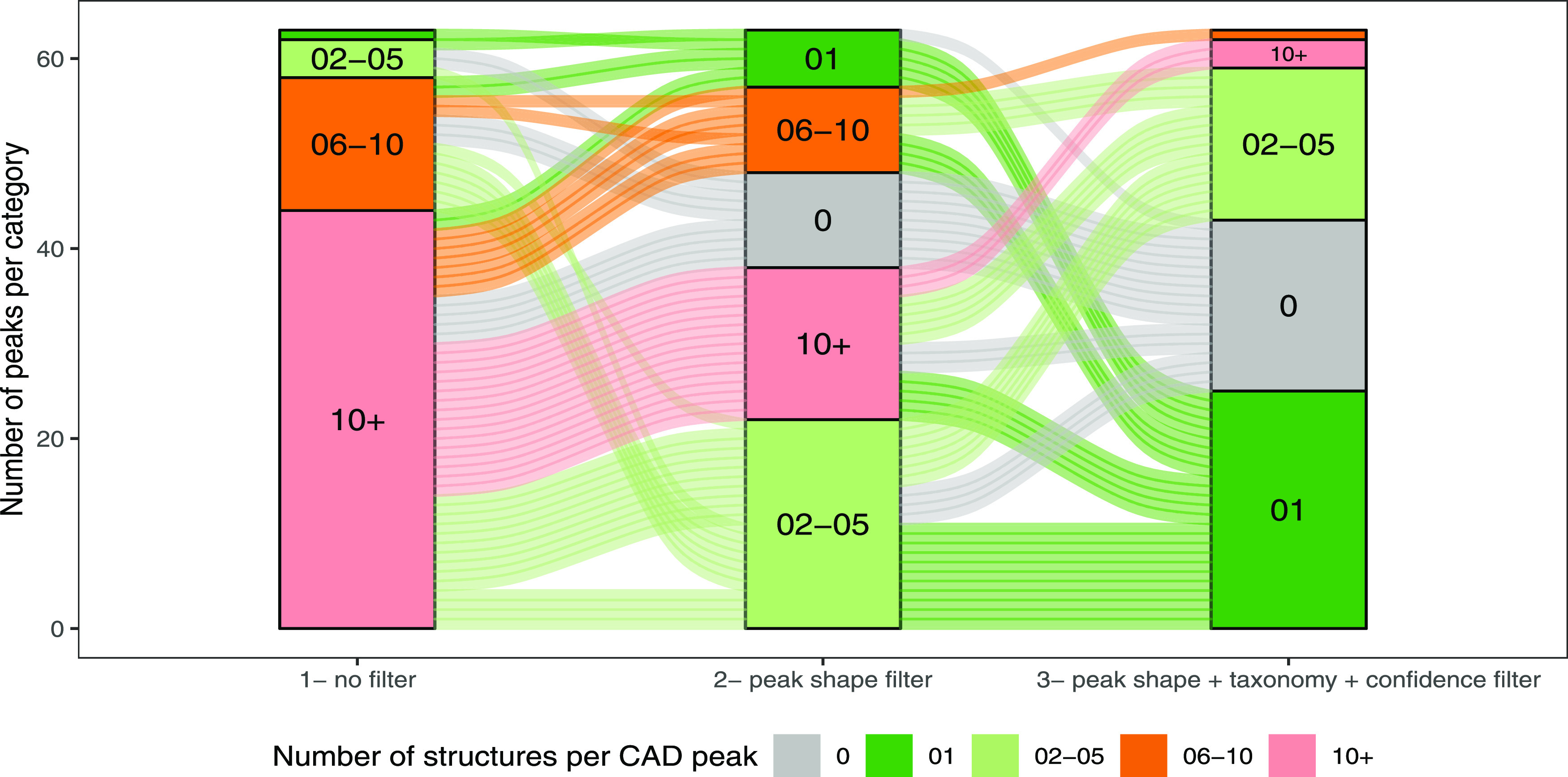
Alluvial plot of the informed data filtering. The underlying data are in the PI mode. The alluvial plot is colored according to five categories. The categories correspond to the number of candidate structures per CAD peak. The first block (from left to right) represents the situation before filtering, the second block after peak-shape filtering, and the last block after peak-shape filtering, taxonomic filter, and annotation confidence filter.

The metrics in Table 1 could also be used to evaluate the CAD peak purity.^51,52^ The peak purity could then be used to evaluate the quality of the acquired fragmentation spectra,^53^ or even try to deconvolute them.^54^ Therefore, to complement the averages shown in Table 1, an alluvial plot summarizing the filtering process applied to the MS features below each CAD peak in the PI data is illustrated in Figure 3.

This stringent filtering allowed one to avoid part of the inconsistent annotations generated by automated annotation approaches and focus on consistent ones only. The filtering not only reduced the number of candidates linked to a CAD peak but also discarded the ones whose annotation was not confident (see gray block in Figure 3). For notice, features not passing the filtering process are kept as belonging to the “minor” metabolome. Starting from a situation where no CAD peak was unambiguously linked to a single structure, almost half of the CAD peaks were unambiguously linked to a structure at the end. Thus, the unambiguous annotation of the major metabolome could be automated for a portion of the peaks. When ambiguity remained, each plausible candidate was kept while considerably simplifying the initial data. In order to make full use of these filtered metabolite profiling data, different ways of visualizing the major and minor metabolome annotations are suggested below.

### Data Visualization

**Figure 4:**
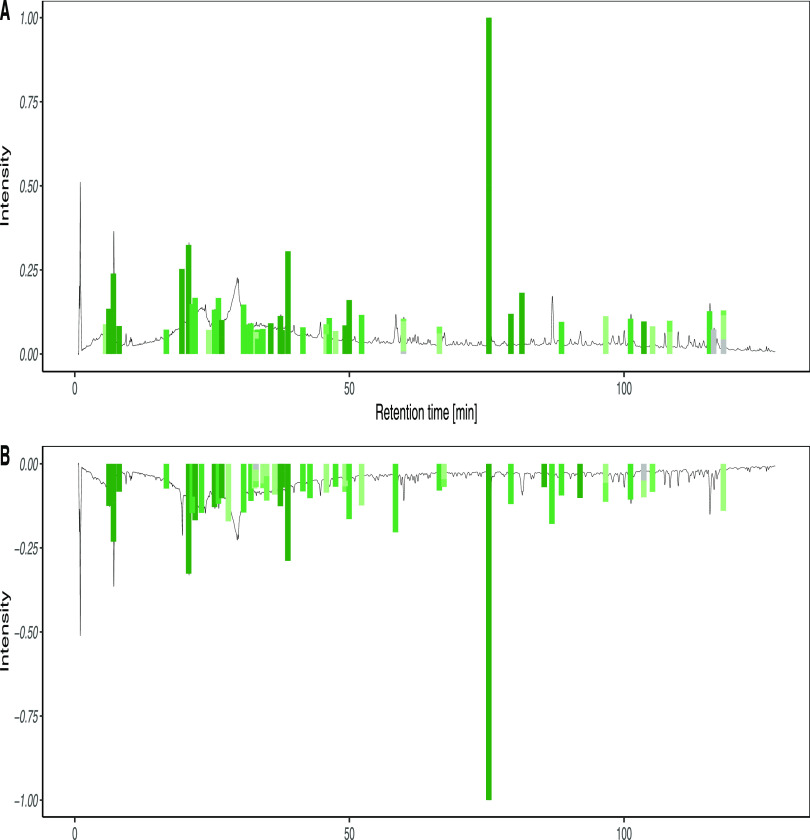
Taxonomically informed pseudochromatograms. The bars are colored according to the taxon level of the closest organism in which the compound was found relative to the biological source of the extract. Dark green bars indicate that the compound was already reported in the species. The remaining categories follow the same logic. Panel A illustrates the annotations in positive ionization mode while panel B illustrates the annotations in negative ionization mode.

**Figure 5:**
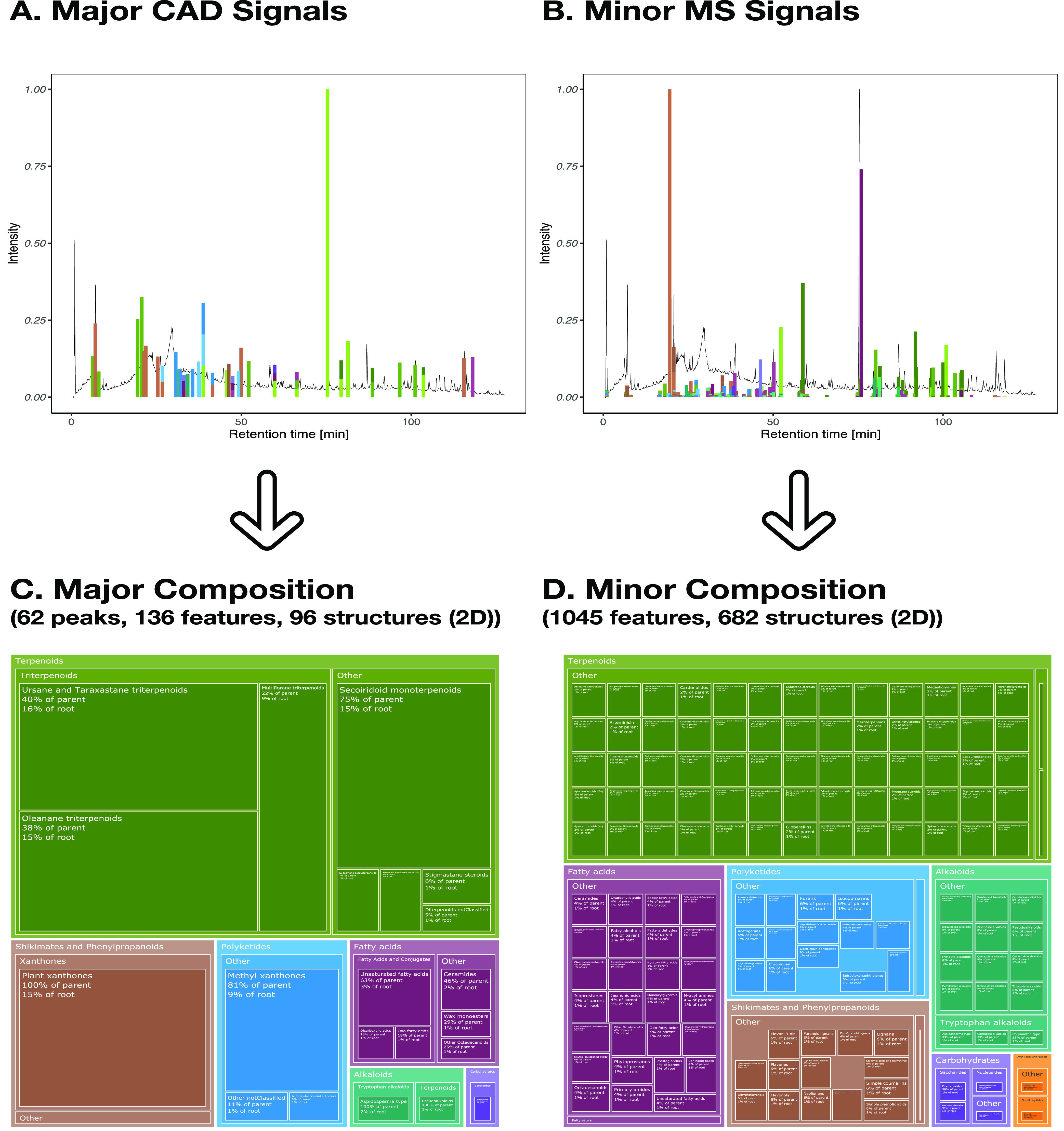
Chemically informed pseudochromatograms and related treemaps. In panels A and B, the bars are colored according to the chemical superclass attributed to the features obtained after filtering. Panel A illustrates the features that were classified as major, while panel B as minor. The data used were the positive ionization mode, and intensities were normalized. In panels C and D, the chemical classes (represented as bars in panels A and B) are regrouped into categories, independently from their position on the chromatogram. For panel C, the area of the rectangle is proportional to the CAD area. For panel D, MS intensity was neglected, and the number of annotations corresponding to a given chemical class is illustrated.

To facilitate further analysis, various visualizations are created using the processed data. One such visualization is the pseudochromatogram, which combines chromatograms with histograms representing the integrated peak area. These pseudochromatograms provide insights into the biological origin of the annotated compounds relative to the biological source of the extract, as illustrated in Figure 4. In both panels, the main CAD peaks are related to the green or dark green categories (darker, meaning closely related organisms). This confirms the logic of favoring compounds already isolated from nearby organisms as being attributed to the CAD peaks. Although a majority of CAD peaks are annotated in both modes, an interesting complementarity can be observed. *S. chirayita* is not a well-studied species, nor is the genus *Swertia*, but the family Gentianaceae is. Therefore, its analysis can benefit from previously acquired data on other closely related organisms. In the case of less studied organisms, belonging to a poorly studied branch of the tree of life, it will be necessary to rely more on chemical class attribution and the confidence of the spectral score. The bars can also be adapted to reflect the NPClassifier chemical pathways (and chemical superclasses) present in the extract.^17^ Then, these bars can be aggregated together, as shown in Figure 5.

Following the methodology described above, the majority of CAD peaks from the *S. chirayita* extract were annotated with structures belonging to the terpenoid pathway, according to the NPClassifier chemical taxonomy. They accounted for 58% of the total area of the peaks (Figure 5, panel C). Terpenoids also represented most of the “minor” signals. The second most important pathways were the shikimates and phenylpropanoids (16%). A fair amount of peaks were also attributed to the polyketide pathway (15%), because of the ambiguous biosynthetic origin of xanthones. In plants, xanthones are known to be produced by the shikimate pathway,^55^ but NPClassifier classified part of them as coming from the polyketide pathway (mainly in Fungi). Fungal endophytes are known to account for many of the NPs found in plants, and also xanthones.^56^ As part of a general trend, the proportion of chemical classes in the minor composition has roughly followed that of the major composition, with the exception of fatty acids and alkaloids, which were overrepresented in the minor metabolome. The disproportional amount of alkaloids detected can be attributed to their good ionization in PI. Such compounds are detected at trace levels and do not yield corresponding CAD peaks. The fatty acids form the basis of membranes. Although the extraction process (see Experimental Section) is designed to remove them, it is difficult to deplete them completely. Moreover, they are essential building blocks for more evolved structures, and observing them as a leftover of plants’ combinatorial biosynthesis is expected.^57^

#### Automated Reporting and Literature Matching

In addition to the pseudochromatograms and treemaps presented above, an automated tabular report with the most relevant information per CAD peak can be generated.^58−88^ An example of such a table is presented in Table 2. The table shows the 65 integrated CAD peaks (from PI and NI), of which 17 were associated with structures previously reported in *S. chirayita*, and 15 additional in other *Swertia* spp. The majority of the annotated structures were xanthones. Three peaks were associated with a structure found in Gentianaceae, and four were in the Gentianales. Two peaks were already found in Magnoliopsida, but with good spectral matching scores, and were thus kept. Finally, one peak annotated as a ceramide by Sirius was kept even if never found in plants, as its initial score was extremely good. Partial overfitting might explain this score above 1 and should be investigated. Finally, 18 CAD peaks remained without confident annotation, accounting for 22.7% of the overall peak area. These peaks should be prioritized for further investigation by experts with the aim of structural elucidation. An additional column highlighting the closest organism from which the compound was isolated is available. The information is retrieved from Wikidata, which is currently the largest resource for NP occurrences.^89^ The studied ethanolic extract of *S. chirayita* underwent a concentration step to remove polar compounds, thus favoring more apolar compounds such as triterpenoids, which represent the most abundant class annotated (see Experimental Section). The annotated triterpenoids correspond to the major red peaks between 75 and 92 min in Figure 1, further reinforcing the reason for using a CAD detector, as the PDA detector would not have detected these compounds.

**Table 2:**
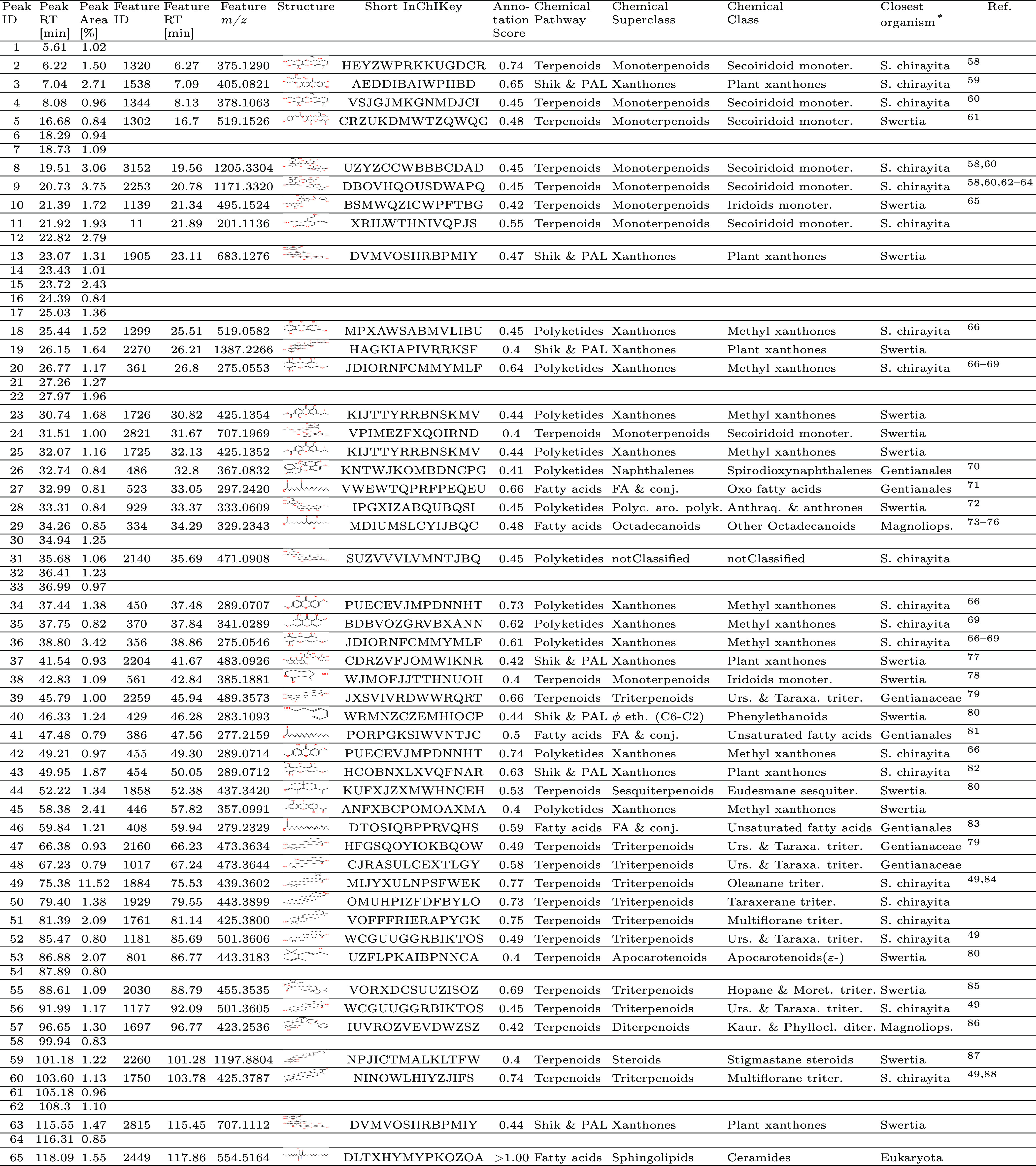
Automated Report of the Major Metabolome of *Swertia chirayita*

a Organism with the shortest taxonomic distance where the structure was reported.

While our efforts were focused on automation of the confidence in the annotation and peak-shape similarity calculation, some improvements in the quantitation could be made. The error in the quantitation induced by the changing eluent composition could be reduced by implementing an inverse gradient, as in ref (34). A reverse gradient involves adding postcolumn solvent so that the composition of the resulting eluent is constant.^90^ Indeed, the proportion of organic solvent has a considerable effect on the CAD response.^91,92^ This could also be corrected by using algorithms, such as those suggested by refs (93 and 94). However, the proportion of organic solvent is not the only parameter for improving the precision and accuracy of the quantitation.^95^ Even if ELSD and CAD are often considered to respond universally, significant differences have been observed depending on the chemical structures studied.^96^ In addition, CAD response is nonlinear and uses a power function that has to be taken into account.^97,98^ The chromatographic resolution is another limitation. Indeed, high peak capacities (number of peaks theoretically separated with a resolution of 1 within a chromatographic run)^99^ are required to achieve good results. While addressing these limitations could improve the quantitative results, our workflow is already useful for generating comprehensive data on the composition of natural extracts, covering minor and major metabolomes.

#### CAD-Informed Molecular Network

**Figure 6:**
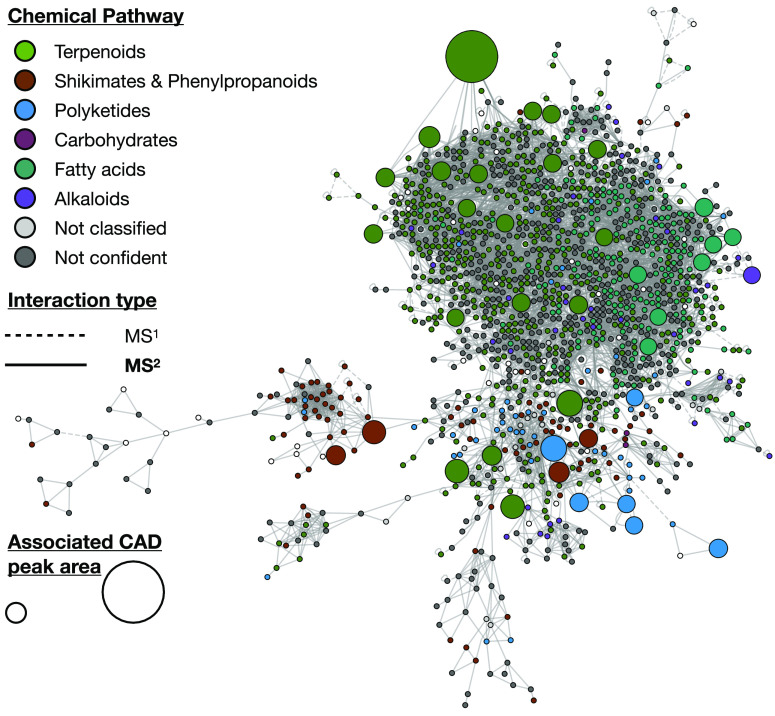
CAD-informed molecular network. Each node represents a feature. Features are colored according to the chemical pathway of their annotation. The size of the nodes corresponds to the peak area of the associated CAD peak. The type of line connecting the nodes corresponds to the interaction used to build the edge. Big- and medium-sized nodes can serve as anchor points for further exploration. Only the largest cluster (in positive ionization mode) is illustrated. The corresponding GNPS job is 36f42cb647c24b0c80e335a44310e27b.

As the workflow presented enables the annotation of peaks associated with the major and minor metabolome, the molecular networks make it easy to link major NPs and their minor analogues. Figure 6 illustrates the largest cluster of the PI analysis. Features were colored according to the chemical pathway of their annotation. By adding the CAD information as the size of the nodes, the molecular network included an additional semiquantitative dimension on the features obtained after filtering (see Table 1). This dimension could be used to target investigations to nodes correlated to major CAD signals or minor analogues that are linked to them. The semiquantitative dimension brought by the CAD is crucial to guide further efforts, as the amount of data can be misleading toward compounds favored only because of their ionization potential. We expect such visualization to facilitate downstream tasks such as targeted isolation and full structural elucidation of the relevant features. This strategy was already used in a collaborative study to target the isolation of photoactive pigments by taking into account some particular UV signals.^100^ While the majority of preparative-scale instruments are hyphenated with UV detectors, the use of more generic detectors such as ELSD or CAD will facilitate the isolation of new NPs without chromophores.

## Experimental Section

### Plant Material

The aerial parts of *S. chirayita* were supplied by Tradall SA (Batch 155174).

### Extraction

The plant material was used for other studies; therefore, high amounts of extract were needed.

1.1 kg of plant material was homogenized in a grinder and extracted at room temperature with 5 kg of H_2_O and 6 kg of EtOH for 4 days. The extract was then filtered and stored in inox for decantation for 10 days. Finally, the extract was concentrated under reduced pressure, freeze-dried, and stored at −20 °C until further use. From 1.1 kg of plant material, 86.3 g of dried extract was obtained.

### Vacuum Liquid Chromatography

Because the extract used for biological experiments was not described here, large amounts were needed. The first fractionation of the extract was undertaken by Vacuum Liquid Chromatography (VLC). 10 g of extract mixed with 20 g of silica was loaded on a chromatographic system made of two layers of silica (50 g and 200 g) separated by sand. This system was first very gently (approximately 2 drops/s) eluted with 3 × 500 mL of 100% H_2_O and then 3 × 500 mL of 100% EtOH. The aqueous and ethanolic parts were collected separately, with a “mix” part corresponding to the dead volume of the system between both and the system was washed with DCM. All VLC fractions were then concentrated under reduced pressure, freeze-dried, and stored at −20 °C until further use. This procedure was repeated 3 times. From the initial 30 g of extract, a total of 27.4 g was recovered (91.3%). The mass of VLC_1 (H_2_O) was 12.4 g, VLC_2 (mix) was 4.2 g, VLC_3 (EtOH) was 10.4 g. and VLC_4 (wash) was 0.3 g.

A small aliquot of VLC_3 resuspended at 5 mg/mL in EtOH was used for the data acquisition.

### Data Acquisition

Chromatographic separation was performed on a Waters Acquity UHPLC system interfaced with a Corona Veo RS Charged Aerosol Detector (CAD) and a Q-Exactive Focus mass spectrometer using a heated electrospray ionization source. Thermo Scientific Xcalibur 3.1 software was used for instrument control. The LC conditions were as follows: column, Waters BEH C_18_ 150 × 2.1 mm, 1.7 μm; mobile phase, (A) water with 0.1% formic acid; (B) acetonitrile with 0.1% formic acid; flow rate, 400 μL·min^–1^; injection volume, 6 μL; gradient, isocratic at 5% B for 0.5 min linear gradient of 5–100% B over 126.5 min (21.5 min for the short run) and isocratic at 100% B for 12 min (2 min for the short run). The source parameters were as follows: source voltage, 3.5 kV (pos); sheath gas flow rate (N_2_), 55 units; auxiliary gas flow rate, 15 units; spare gas flow rate, 3.0; capillary temperature, 350.00 °C; and S-Lens RF Level, 45. The mass analyzer was calibrated using a mixture of caffeine, methionine–arginine–phenylalanine–alanine–acetate, sodium dodecyl sulfate, sodium taurocholate, and Ultramark 1621 in an acetonitrile/methanol/water solution containing 1% formic acid by direct injection. The data-dependent MS/MS events were performed on the three most intense ions detected in full-scan MS (Top3 experiment). The MS/MS isolation window width was 1 Da, and the stepped normalized collision energy (NCE) was set to 15, 30, and 45 units. In data-dependent MS/MS experiments, full scans were acquired at a resolution of 35,000 fwhm (at *m*/*z* 200) and MS/MS scans at 17,500 fwhm both with an automatically determined maximum injection time. After being acquired in an MS/MS scan, parent ions were placed in a dynamic exclusion list for 2.0 s. A custom exclusion list was used. An Acquity UHPLC PDA detector was used to acquire UV spectra, which were detected from 200 to 500 nm. An analytical split was used with a split ratio of 9:1 (CAD/MS). CAD parameters were: evaporation temperature at 40 °C, 5 bar N_2_, power function 1.

### Data Conversion

All raw data files were converted using ThermoRawFileParser v.1.4.0.^101^ Currently, only ThermoRawFileParser is available to encode the CAD signal in the mzML file directly from the raw file (with the --allDetectors option). This feature was added by the authors of the tool on our request (see https://github.com/compomics/ThermoRawFileParser/pull/87 and https://github.com/compomics/ThermoRawFileParser/issues/60). Once converted, the mzML file contained all requested signals with their related chromatograms and spectra. The chromatograms were encoded as BasePeak_0 for the BPI chromatogram, PDA.1_TotalAbsorbance_0 for the PDA chromatogram, and UV.1_CAD_1_0 for the CAD chromatogram. The generic command used was: monoThermoRawFileParser.exe-d $DIRECTORY--allDetectors--format = 2.

### Data Processing

#### Signals’ Pretreatment

A manual investigation after the coupling of the different detectors to the LC measured that the MS signal was delayed by 0.090 and 0.055 min in comparison to the one of the PDA and the CAD, respectively. These values were used to align the signals.

To reduce the noise, signals underwent a Fourier transform. To perform the Fourier transform, the filterFFT function from the nucleR package^102^ was used. Fourier components were set to 0.01. Further signal sharpening was performed as described in ref (37) with the implementation of the function in R (see https://github.com/Adafede/cascade/blob/main/R/signal_sharpening.R).

#### Charged Aerosol Detector Data

Peaks were detected and integrated using the get_peaks function from the chromatographR package.^103^ Default parameters were used.

#### MS Features’ Extraction and Ion Identity Networking

MS features were extracted and informed using MZmine3 v.3.4.0.^104^ All parameters were given in the form of an .xml file using the batch mode. The details of the xml file are available in the Appendix. The template xml file was filled with filename and MS mode using a bash command available in the Appendix. Adducts used for the different Ion Identity Networking steps are included in the file.

#### Feature-Based Molecular Network

In order to keep the retention time, the exact mass information and to allow for the separation of isomers, a feature-based molecular network (https://ccms-ucsd.github.io/GNPSDocumentation/featurebasedmolecularnetworking/) was created using the .mgf file resulting from the MZMine pretreatment step detailed above. When the parameter was not specified, default parameters were used. A network was then created where edges were filtered to have a cosine score above 0.6 and more than six matched peaks. Further edges (MS^2^ relationships) between the two nodes were kept in the network if and only if each of the nodes appeared in each other’s respective top 20 most similar nodes. The maximum shift between precursors was set to 500 Da. The maximum component size was set to 0. Edges (MS^2^ or MS^1^ relationships) were added as supplementary pairs. The spectra in the network were then searched against the GNPS’ spectral libraries. All matches kept between network and library spectra were required to have a score above 0.7 and at least six matched peaks. The output was visualized using Cytoscape 3.9.1 software.^105^ The GNPS job parameters and resulting data are available at the following addresses: 36f42cb647c24b0c80e335a44310e27b for positive and 8c2a1c97698e420d871c70bfdc80940a for negative.

#### MS Features’ Annotation

##### Sirius

Sirius annotations were performed in batch mode by using Sirius 5.6.3. The details of the commands used for submission are available in the Appendix.

##### GNPS

GNPS annotations were retrieved from the following jobs:

- PI: 2a49fa45a5a74a8794399619d07359b5,
- NI: ef4b9a47a97c424d8b9411f09018a8e7,
- PI enriched: 2621c3e3af7e48298433fca269d8d1cb,
- NI enriched: 7066d9d60e694957a4798fe3a990a35e,
- PI enriched long: 36f42cb647c24b0c80e335a44310e27b,
- NI enriched long: 8c2a1c97698e420d871c70bfdc80940a

##### ISDB

The in silico libraries in positive and negative modes were generated as described in ref (47). The SMILES used were from ref (106) CFM 4.0 was used for in silico fragmentation.^107^ Parameters are available at https://github.com/mandelbrot-project/spectral_lib_builder. In silico spectral matching was performed using spectral_lib_matcher, based on the matchms library.^108^ Parent mass and MS/MS tolerances were 0.01 Da, the similarity method used ModifedCosine, and the minimum score and minimum peaks set to 0.

##### Taxonomically Informed Metabolite Annotation

Taxonomically Informed Metabolite Annotation was performed using all the above-mentioned inputs. It was performed using TIMA v2.8.0.^20,43^ All parameters were the default ones, archived at. An up-to-date version of the documentation is available at https://taxonomicallyinformedannotation.github.io/tima-r/.

Annotations were considered confident when the final score after TIMA was above 0.4.

#### Link Between CAD and MS Data

The extracted MS feature list was reused in R to extract exact MS peak shapes and compare them to the CAD. Both chromatograms were resampled to the same frequency of 2 Hz. Therefore, peak intensity was sampled from the extrapolated traces each 0.5 s. Peak shapes were then compared using the compareChromatograms function from the MSnbase package with the closest method as an argument.^109,110^

### Literature Matching

Compounds were retrieved from Wikidata as described in ref (89). The template of the query used is available at https://github.com/Adafede/wd-np-up-to-date/. Only references published between 1900 and 2023 were kept. Only compounds with an MW between 150 and 1500 Da and an xlogP between −1 and 6 were kept.

## Data Availability

The data are available through the GNPS job IDs (PI: 2a49fa45a5a74a8794399619d07359b5, NI: ef4b9a47a97c424d8b9411f09018a8e7, PI enriched: 2621c3e3af7e48298433fca269d8d1cb, NI enriched: 7066d9d60e694957a4798fe3a990a35e, PI enriched long: 36f42cb647c24b0c80e335a44310e27b, NI enriched long: 8c2a1c97698e420d871c70bfdc80940a). All programs written for this work can be found in the following repository: https://github.com/Adafede/cascade. The main dependencies were described before.

## Supporting Information Available

Supporting Information 1. Comparison of peak detection on the raw and processed signal; Supporting Information 2. MS features-CAD peaks statistics (Negative Ionization); Supporting Information 3. MZmine commands used: both positive and negative; Supporting Information 4. Sirius commands used: both positive and negative (PDF)
