# Supplementary material for: Automated Composition Assessment of Natural Extracts: Untargeted Mass Spectrometry-Based Metabolite Profiling Integrating Semiquantitative Detection
Source: J Agric Food Chem. 2023 Nov 10;71(46):18010–23. doi: 10.1021/acs.jafc.3c03099 (PMC10683005; doi:10.1021/acs.jafc.3c03099)
Supplement: Supplementary file 1 — jf3c03099_si_001.pdf [file jf3c03099_si_001.pdf]

Automated Composition Assessment of Natural Extracts: Untargeted Mass Spectrometry-Based Metabolite Profiling Integrating Semi-quantitative Detection

Adriano Rutz *,†,‡,¶ and Jean-Luc Wolfender *,†,‡

†School of Pharmaceutical Sciences, University of Geneva, 1211 Geneva, Switzerland

‡Institute of Pharmaceutical Sciences of Western Switzerland, University of Geneva, 1211 Geneva, Switzerland

¶Institute of Molecular Systems Biology, ETH Zürich, 8093 Zürich, Switzerland

E-mail: rutz@imsb.biol.ethz.ch; jean-luc.wolfender@unige.ch

Supporting Information 1. Comparison of peak detection on the raw and processed signal

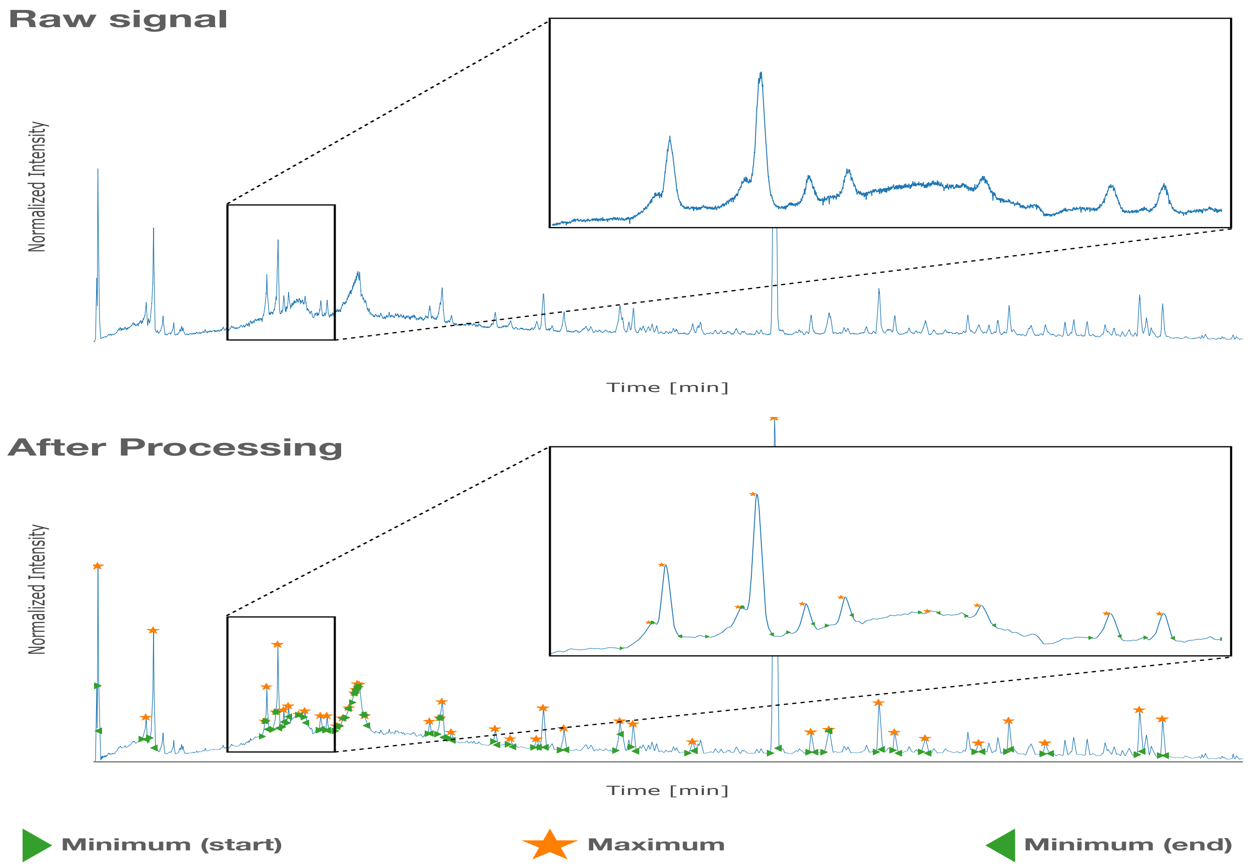

Figure 1: **Comparison of peak detection on the raw and processed signal.** The result of the automated peak picking on the raw signal is shown on the top. No peaks are detected due to the noisy signal. Peak detection and integration on the processed signal is shown at the bottom. Almost no peak is lost and only poorly-shaped peaks are split. A zoomed-in area showing more into detail the signal differences is also illustrated.

Peak picking was performed using the `chromatographR` package.[1] The result is illustrated in Figure 1. While no peaks were integrated automatically before processing (top), the majority of the visible peaks were correctly integrated (bottom). Very small, poorly defined peaks were not picked, while major peaks were. Even if manual integration of such data would perform better, automated pick peaking is still a challenge, as illustrated by diverse recent efforts trying to include machine learning to solve it.[2,3]

Supporting Information 2. MS features-CAD peaks statistics (negative ionization)

Table 1: MS features-CAD peaks statistics (Negative Ionization)

	**No filter**	**Peak-shape filter**	**Peak-shape filter and taxo filter**
Picked features	2,276	2,276	2,276
Picked CAD peaks	62	62	62
Features linked to CAD peak	887	290	110
Number of features per peak *	14.3	5.8	2.5
Number of structures per peak *	11.6	4.9	1.9
Number of MFs per peak *	11.6	4.9	1.9
Number of chemical classes per peak *	9.7	4.5	1.9

* On average

Supporting Information 3. MZmine commands used (positive and negative

3.1 MZmine template (positive)


```xml
<?xml version="1.0" encoding="UTF-8"?><batch mzmine_version="3.4.0">
    <batchstep method="io.github.mzmine.modules.io.import_rawdata_all.AllSpectralDataImportModule" parameter_version="1">
        <parameter name="File names">
            <file>MYFILEPATH</file>
        </parameter>
        <parameter name="Advanced import" selected="false">
            <parameter name="MS1 detector (Advanced)" selected="false" selected_item="Factor of lowest signal">
                <module name="Factor of lowest signal">
                    <parameter name="Noise factor">2.5</parameter>
                </module>
                <module name="Centroid">
                    <parameter name="Noise level">0.0</parameter>
                    <parameter name="Detect isotope signals below noise level" selected="true">
                        <parameter name="Chemical elements">H,C,N,O,S,P</parameter>
                        <parameter name="m/z tolerance">
                            <absolutetolerance>0.0</absolutetolerance>
                            <ppmtolerance>4.0</ppmtolerance>
                        </parameter>
                        <parameter name="Maximum charge of isotope m/z">2</parameter>
                    </parameter>
                </module>
                <module name="Exact mass">
                    <parameter name="Noise level"/>
                    <parameter name="Detect isotope signals below noise level" selected="true">
                        <parameter name="Chemical elements">H,C,N,O,S,P</parameter>
                        <parameter name="m/z tolerance">
                            <absolutetolerance>0.0</absolutetolerance>
                            <ppmtolerance>4.0</ppmtolerance>
                        </parameter>
                        <parameter name="Maximum charge of isotope m/z">2</parameter>
                    </parameter>
                </module>
                <module name="Local maxima">
                    <parameter name="Noise level"/>
                </module>
                <module name="Recursive threshold">
                    <parameter name="Noise level"/>
                    <parameter name="Min m/z peak width"/>
                    <parameter name="Max m/z peak width"/>
                </module>
                <module name="Wavelet transform">
                    <parameter name="Noise level"/>
                    <parameter name="Scale level"/>
                    <parameter name="Wavelet window size (%)"/>
                </module>
                <module name="Auto">
                    <parameter name="Noise level">100000.0</parameter>
                    <parameter name="Detect isotope signals below noise level" selected="true">
                        <parameter name="Chemical elements">H,C,N,O,S,P</parameter>
                        <parameter name="m/z tolerance">
                            <absolutetolerance>0.0</absolutetolerance>
                            <ppmtolerance>4.0</ppmtolerance>
                        </parameter>
                        <parameter name="Maximum charge of isotope m/z">2</parameter>
                    </parameter>
                </module>
            </parameter>
            <parameter name="MS2 detector (Advanced)" selected="false" selected_item="Factor of lowest signal">
                <module name="Factor of lowest signal">
                    <parameter name="Noise factor">2.5</parameter>
                </module>
                <module name="Centroid">
                    <parameter name="Noise level">0.0</parameter>
                    <parameter name="Detect isotope signals below noise level" selected="true">
                        <parameter name="Chemical elements">H,C,N,O,S,P</parameter>
                        <parameter name="m/z tolerance">
                            <absolutetolerance>0.0</absolutetolerance>
                            <ppmtolerance>4.0</ppmtolerance>
                        </parameter>
                        <parameter name="Maximum charge of isotope m/z">2</parameter>
                    </parameter>
                </module>
                <module name="Exact mass">
                    <parameter name="Noise level"/>
                    <parameter name="Detect isotope signals below noise level" selected="true">
                        <parameter name="Chemical elements">H,C,N,O,S,P</parameter>
                        <parameter name="m/z tolerance">
                            <absolutetolerance>0.0</absolutetolerance>
                            <ppmtolerance>4.0</ppmtolerance>
```

```
                </parameter>
                <parameter name="Maximum charge of isotope m/z">2</parameter>
            </parameter>
        </module>
        <module name="Local maxima">
            <parameter name="Noise level"/>
        </module>
        <module name="Recursive threshold">
            <parameter name="Noise level"/>
            <parameter name="Min m/z peak width"/>
            <parameter name="Max m/z peak width"/>
        </module>
        <module name="Wavelet transform">
            <parameter name="Noise level"/>
            <parameter name="Scale level"/>
            <parameter name="Wavelet window size (%)"/>
        </module>
        <module name="Auto">
            <parameter name="Noise level">0.0</parameter>
            <parameter name="Detect isotope signals below noise level" selected="true">
                <parameter name="Chemical elements">H,C,N,O,S,P</parameter>
                <parameter name="m/z tolerance">
                    <absolutetolerance>0.0</absolutetolerance>
                    <ppmtolerance>4.0</ppmtolerance>
                </parameter>
                <parameter name="Maximum charge of isotope m/z">2</parameter>
            </parameter>
        </module>
    </parameter>
    <parameter name="Denormalize fragment scans (traps)">false</parameter>
  </parameter>
  <parameter name="Spectral library files"/>
</batchstep>
<batchstep method="io.github.mzmine.modules.dataprocessing.featdet_massdetection.MassDetectionModule" parameter_version="1">
  <parameter name="Raw data files" type="BATCH_LAST_FILES"/>
  <parameter name="Scan filters" selected="true">
    <parameter name="Scan number"/>
    <parameter name="Base Filtering Integer"/>
    <parameter name="Retention time"/>
    <parameter name="Mobility"/>
    <parameter name="MS level filter" selected="MS2, level = 2">2</parameter>
    <parameter name="Scan definition"/>
    <parameter name="Polarity">Any</parameter>
    <parameter name="Spectrum type">ANY</parameter>
  </parameter>
  <parameter name="Scan types (IMS)">All scan types</parameter>
  <parameter name="Mass detector" selected_item="Auto">
    <module name="Factor of lowest signal">
        <parameter name="Noise factor">2.5</parameter>
    </module>
    <module name="Centroid">
        <parameter name="Noise level">0.0</parameter>
        <parameter name="Detect isotope signals below noise level" selected="true">
            <parameter name="Chemical elements">H,C,N,O,S,P</parameter>
            <parameter name="m/z tolerance">
                <absolutetolerance>0.0</absolutetolerance>
                <ppmtolerance>4.0</ppmtolerance>
            </parameter>
            <parameter name="Maximum charge of isotope m/z">2</parameter>
        </parameter>
    </module>
    <module name="Exact mass">
        <parameter name="Noise level"/>
        <parameter name="Detect isotope signals below noise level" selected="true">
            <parameter name="Chemical elements">H,C,N,O,S,P</parameter>
            <parameter name="m/z tolerance">
                <absolutetolerance>0.0</absolutetolerance>
                <ppmtolerance>4.0</ppmtolerance>
            </parameter>
            <parameter name="Maximum charge of isotope m/z">2</parameter>
        </parameter>
    </module>
    <module name="Local maxima">
        <parameter name="Noise level"/>
    </module>
    <module name="Recursive threshold">
        <parameter name="Noise level"/>
        <parameter name="Min m/z peak width"/>
        <parameter name="Max m/z peak width"/>
    </module>
    <module name="Wavelet transform">
        <parameter name="Noise level"/>
        <parameter name="Scale level"/>
        <parameter name="Wavelet window size (%)"/>
    </module>
    <module name="Auto">
        <parameter name="Noise level">10000.0</parameter>
        <parameter name="Detect isotope signals below noise level" selected="false">
            <parameter name="Chemical elements">H,C,N,O,S,P</parameter>
            <parameter name="m/z tolerance">
                <absolutetolerance>0.0</absolutetolerance>
                <ppmtolerance>4.0</ppmtolerance>
            </parameter>
            <parameter name="Maximum charge of isotope m/z">2</parameter>
        </parameter>
    </module>
  </parameter>
```

```
    <parameter name="Denormalize fragment scans (traps)">false</parameter>
    <parameter name="Output netCDF filename (optional)" selected="false"/>
</batchstep>
<batchstep method="io.github.mzmine.modules.dataprocessing.featdet_massdetection.MassDetectionModule" parameter_version="1">
    <parameter name="Raw data files" type="BATCH_LAST_FILES"/>
    <parameter name="Scan filters" selected="true">
        <parameter name="Scan number"/>
        <parameter name="Base Filtering Integer"/>
        <parameter name="Retention time"/>
        <parameter name="Mobility"/>
        <parameter name="MS level filter" selected="MS1, level = 1">1</parameter>
        <parameter name="Scan definition"/>
        <parameter name="Polarity">Any</parameter>
        <parameter name="Spectrum type">ANY</parameter>
    </parameter>
    <parameter name="Scan types (IMS)">All scan types</parameter>
    <parameter name="Mass detector" selected_item="Auto">
        <module name="Factor of lowest signal">
            <parameter name="Noise factor">2.5</parameter>
        </module>
        <module name="Centroid">
            <parameter name="Noise level">0.0</parameter>
            <parameter name="Detect isotope signals below noise level" selected="true">
                <parameter name="Chemical elements">H,C,N,O,S,P</parameter>
                <parameter name="m/z tolerance">
                    <absolutetolerance>0.0</absolutetolerance>
                    <ppmtolerance>4.0</ppmtolerance>
                </parameter>
                <parameter name="Maximum charge of isotope m/z">2</parameter>
            </parameter>
        </module>
        <module name="Exact mass">
            <parameter name="Noise level"/>
            <parameter name="Detect isotope signals below noise level" selected="true">
                <parameter name="Chemical elements">H,C,N,O,S,P</parameter>
                <parameter name="m/z tolerance">
                    <absolutetolerance>0.0</absolutetolerance>
                    <ppmtolerance>4.0</ppmtolerance>
                </parameter>
                <parameter name="Maximum charge of isotope m/z">2</parameter>
            </parameter>
        </module>
        <module name="Local maxima">
            <parameter name="Noise level"/>
        </module>
        <module name="Recursive threshold">
            <parameter name="Noise level"/>
            <parameter name="Min m/z peak width"/>
            <parameter name="Max m/z peak width"/>
        </module>
        <module name="Wavelet transform">
            <parameter name="Noise level"/>
            <parameter name="Scale level"/>
            <parameter name="Wavelet window size (%)"/>
        </module>
        <module name="Auto">
            <parameter name="Noise level">0.0</parameter>
            <parameter name="Detect isotope signals below noise level" selected="true">
                <parameter name="Chemical elements">H,C,N,O,S,P</parameter>
                <parameter name="m/z tolerance">
                    <absolutetolerance>0.0</absolutetolerance>
                    <ppmtolerance>4.0</ppmtolerance>
                </parameter>
                <parameter name="Maximum charge of isotope m/z">2</parameter>
            </parameter>
        </module>
    </parameter>
    <parameter name="Denormalize fragment scans (traps)">false</parameter>
    <parameter name="Output netCDF filename (optional)" selected="false"/>
</batchstep>
<batchstep method="io.github.mzmine.modules.dataprocessing.featdet_adapchromatogrambuilder.ModularADAPChromatogramBuilderModule"
      parameter_version="1">
    <parameter name="Raw data files" type="BATCH_LAST_FILES"/>
    <parameter name="Scan filters" selected="true">
        <parameter name="Scan number"/>
        <parameter name="Base Filtering Integer"/>
        <parameter name="Retention time">
            <min>0.0</min>
            <max>150.0</max>
        </parameter>
        <parameter name="Mobility"/>
        <parameter name="MS level filter" selected="MS1, level = 1">1</parameter>
        <parameter name="Scan definition"/>
        <parameter name="Polarity">Any</parameter>
        <parameter name="Spectrum type">ANY</parameter>
    </parameter>
    <parameter name="Minimum consecutive scans">4</parameter>
    <parameter name="Minimum intensity for consecutive scans">10000.0</parameter>
    <parameter name="Minimum absolute height">50000.0</parameter>
    <parameter name="m/z tolerance (scan-to-scan)">
        <absolutetolerance>0.0</absolutetolerance>
        <ppmtolerance>12.0</ppmtolerance>
    </parameter>
    <parameter name="Suffix">eics</parameter>
    <parameter name="Allow single scan chromatograms"/>
</batchstep>
<batchstep method="io.github.mzmine.modules.dataprocessing.featdet_smoothing.SmoothingModule" parameter_version="1">
```

```xml
    <parameter name="Feature lists" type="BATCH_LAST_FEATURELISTS"/>
    <parameter name="Smoothing algorithm" selected_item="Savitzky Golay">
        <module name="Savitzky Golay">
            <parameter name="Retention time smoothing" selected="true">5</parameter>
            <parameter name="Mobility smoothing" selected="false">5</parameter>
        </module>
        <module name="Loess smoothing">
            <parameter name="Retention time width (scans)" selected="false">5</parameter>
            <parameter name="Mobility width (scans)" selected="false">5</parameter>
        </module>
    </parameter>
    <parameter name="Original feature list">REMOVE</parameter>
    <parameter name="Suffix">sm</parameter>
</batchstep>
<batchstep method="io.github.mzmine.modules.dataprocessing.featdet_chromatogramdeconvolution.minimumsearch.MinimumSearchFeatureResolverModule"
       parameter_version="2">
    <parameter name="Feature lists" type="BATCH_LAST_FEATURELISTS"/>
    <parameter name="Suffix">r</parameter>
    <parameter name="Original feature list">REMOVE</parameter>
    <parameter name="MS/MS scan pairing" selected="true">
        <parameter name="Minimum relative feature height" selected="true">0.15</parameter>
        <parameter name="Minimum required signals" selected="true">1</parameter>
        <parameter name="MS1 to MS2 precursor tolerance (m/z)">
            <absolutetolerance>0.0</absolutetolerance>
            <ppmtolerance>12.0</ppmtolerance>
        </parameter>
        <parameter name="Retention time filter" selected="Use feature edges" unit="MINUTES">0.05</parameter>
        <parameter name="Limit by ion mobility edges">true</parameter>
        <parameter name="Merge MS/MS spectra (TIMS)">false</parameter>
        <parameter name="Minimum signal intensity (absolute, TIMS)" selected="false">6000.0</parameter>
        <parameter name="Minimum signal intensity (relative, TIMS)" selected="false">0.1</parameter>
    </parameter>
    <parameter name="Dimension">Retention time</parameter>
    <parameter name="Chromatographic threshold">0.9</parameter>
    <parameter name="Minimum search range RT/Mobility (absolute)">0.05</parameter>
    <parameter name="Minimum relative height">0.0</parameter>
    <parameter name="Minimum absolute height">100000.0</parameter>
    <parameter name="Min ratio of peak top/edge">1.8</parameter>
    <parameter name="Peak duration range (min/mobility)">
        <min>0.0</min>
        <max>1.5</max>
    </parameter>
    <parameter name="Minimum scans (data points)">5</parameter>
</batchstep>
<batchstep method="io.github.mzmine.modules.dataprocessing.filter_isotopegrouper.IsotopeGrouperModule" parameter_version="1">
    <parameter name="Feature lists" type="BATCH_LAST_FEATURELISTS"/>
    <parameter name="Name suffix">deiso</parameter>
    <parameter name="m/z tolerance (intra-sample)">
        <absolutetolerance>0.0</absolutetolerance>
        <ppmtolerance>4.0</ppmtolerance>
    </parameter>
    <parameter name="Retention time tolerance" unit="MINUTES">0.04</parameter>
    <parameter name="Mobility tolerance" selected="false">1.0</parameter>
    <parameter name="Monotonic shape">true</parameter>
    <parameter name="Maximum charge">2</parameter>
    <parameter name="Representative isotope">Most intense</parameter>
    <parameter name="Never remove feature with MS2">true</parameter>
    <parameter name="Original feature list">PROCESS IN PLACE</parameter>
</batchstep>
<batchstep method="io.github.mzmine.modules.dataprocessing.filter_isotopefinder.IsotopeFinderModule" parameter_version="1">
    <parameter name="Feature lists" type="BATCH_LAST_FEATURELISTS"/>
    <parameter name="Chemical elements">H,C,N,O,S,P</parameter>
    <parameter name="m/z tolerance (feature-to-scan)">
        <absolutetolerance>0.0</absolutetolerance>
        <ppmtolerance>4.0</ppmtolerance>
    </parameter>
    <parameter name="Maximum charge of isotope m/z">2</parameter>
    <parameter name="Search in scans">SINGLE MOST INTENSE</parameter>
</batchstep>
<batchstep method="io.github.mzmine.modules.dataprocessing.align_join.JoinAlignerModule" parameter_version="1">
    <parameter name="Feature lists" type="BATCH_LAST_FEATURELISTS"/>
    <parameter name="Feature list name">Aligned feature list</parameter>
    <parameter name="m/z tolerance (sample-to-sample)">
        <absolutetolerance>0.0</absolutetolerance>
        <ppmtolerance>6.0</ppmtolerance>
    </parameter>
    <parameter name="Weight for m/z">3.0</parameter>
    <parameter name="Retention time tolerance" unit="MINUTES">0.15</parameter>
    <parameter name="Weight for RT">1.0</parameter>
    <parameter name="Mobility tolerance" selected="false">1.0</parameter>
    <parameter name="Mobility weight">1.0</parameter>
    <parameter name="Require same charge state">false</parameter>
    <parameter name="Require same ID">false</parameter>
    <parameter name="Compare isotope pattern" selected="false">
        <parameter name="Isotope m/z tolerance">
            <absolutetolerance>0.0</absolutetolerance>
            <ppmtolerance>6.0</ppmtolerance>
        </parameter>
        <parameter name="Minimum absolute intensity"/>
        <parameter name="Minimum score"/>
    </parameter>
    <parameter name="Compare spectra similarity" selected="false">
        <parameter name="Spectral m/z tolerance">
            <absolutetolerance>0.0</absolutetolerance>
            <ppmtolerance>12.0</ppmtolerance>
        </parameter>
```

```
            <parameter name="MS level">2</parameter>
            <parameter name="Compare spectra similarity" selected_item="Weighted cosine similarity">
                <module name="Weighted cosine similarity">
                    <parameter name="Weights">MassBank (mz^2 * I^0.5)</parameter>
                    <parameter name="Minimum cos similarity">0.7</parameter>
                    <parameter name="Handle unmatched signals">KEEP ALL AND MATCH TO ZERO</parameter>
                </module>
                <module name="Composite cosine identity (e.g., GC-EI-MS; similar to NIST search)">
                    <parameter name="Weights">MassBank (mz^2 * I^0.5)</parameter>
                    <parameter name="Minimum cos similarity">0.7</parameter>
                    <parameter name="Handle unmatched signals">KEEP ALL AND MATCH TO ZERO</parameter>
                </module>
            </parameter>
        </parameter>
        <parameter name="Original feature list">REMOVE</parameter>
    </batchstep>
    <batchstep method="io.github.mzmine.modules.dataprocessing.filter_rowsfilter.RowsFilterModule" parameter_version="2">
        <parameter name="Feature lists" type="BATCH_LAST_FEATURELISTS"/>
        <parameter name="Name suffix">13C peak</parameter>
        <parameter name="Minimum aligned features (samples)" selected="false">
            <abs>1</abs>
            <rel>0.0</rel>
        </parameter>
        <parameter name="Minimum features in an isotope pattern" selected="false">2</parameter>
        <parameter name="Validate 13C isotope pattern" selected="true">
            <parameter name="m/z tolerance">
                <absolutetolerance>0.0</absolutetolerance>
                <ppmtolerance>4.0</ppmtolerance>
            </parameter>
            <parameter name="Max charge">2</parameter>
            <parameter name="Estimate minimum carbon">true</parameter>
            <parameter name="Remove if 13C">true</parameter>
            <parameter name="Exclude isotopes">O</parameter>
        </parameter>
        <parameter name="Remove redundant isotope rows">false</parameter>
        <parameter name="m/z" selected="false"/>
        <parameter name="Retention time" selected="false"/>
        <parameter name="features duration range" selected="false">
            <min>0.0</min>
            <max>3.0</max>
        </parameter>
        <parameter name="Chromatographic FWHM" selected="false">
            <min>0.0</min>
            <max>1.0</max>
        </parameter>
        <parameter name="Charge" selected="false">
            <min>1</min>
            <max>2</max>
        </parameter>
        <parameter name="Kendrick mass defect" selected="false">
            <parameter name="Kendrick mass defect">
                <min>0.0</min>
                <max>1.0</max>
            </parameter>
            <parameter name="Kendrick mass base"/>
            <parameter name="Shift">0.0</parameter>
            <parameter name="Charge">1</parameter>
            <parameter name="Divisor">1</parameter>
            <parameter name="Use Remainder of Kendrick mass">false</parameter>
        </parameter>
        <parameter name="Parameter">No parameters defined</parameter>
        <parameter name="Only identified?">false</parameter>
        <parameter name="Text in identity" selected="false"/>
        <parameter name="Text in comment" selected="false"/>
        <parameter name="Keep or remove rows">Keep rows that match all criteria</parameter>
        <parameter name="Feature with MS2 scan">true</parameter>
        <parameter name="Never remove feature with MS2">false</parameter>
        <parameter name="Reset the feature number ID">true</parameter>
        <parameter name="Mass defect" selected="false"/>
        <parameter name="Original feature list">PROCESS IN PLACE</parameter>
    </batchstep>
    <batchstep method="io.github.mzmine.modules.dataprocessing.gapfill_peakfinder.multithreaded.MultiThreadPeakFinderModule" parameter_version="1">
        <parameter name="Feature lists" type="BATCH_LAST_FEATURELISTS"/>
        <parameter name="Name suffix">gaps</parameter>
        <parameter name="Intensity tolerance">0.2</parameter>
        <parameter name="m/z tolerance (sample-to-sample)">
            <absolutetolerance>0.0</absolutetolerance>
            <ppmtolerance>12.0</ppmtolerance>
        </parameter>
        <parameter name="Retention time tolerance" unit="MINUTES">0.1</parameter>
        <parameter name="Minimum scans (data points)">1</parameter>
        <parameter name="Original feature list">PROCESS IN PLACE</parameter>
    </batchstep>
    <batchstep method="io.github.mzmine.modules.dataprocessing.filter_duplicatefilter.DuplicateFilterModule" parameter_version="1">
        <parameter name="Feature lists" type="BATCH_LAST_FEATURELISTS"/>
        <parameter name="Name suffix">dup</parameter>
        <parameter name="Filter mode">NEW AVERAGE</parameter>
        <parameter name="m/z tolerance">
            <absolutetolerance>0.0</absolutetolerance>
            <ppmtolerance>3</ppmtolerance>
        </parameter>
        <parameter name="RT tolerance" unit="MINUTES">0.035</parameter>
        <parameter name="Mobility tolerance" selected="true">0.008</parameter>
        <parameter name="Require same identification">false</parameter>
        <parameter name="Original feature list">PROCESS IN PLACE</parameter>
    </batchstep>
```

```
<batchstep method="io.github.mzmine.modules.dataprocessing.group_metacorrelate.corrgrouping.CorrelateGroupingModule" parameter_version="2">
    <parameter name="Feature lists" type="BATCH_LAST_FEATURELISTS"/>
    <parameter name="RT tolerance" unit="MINUTES">0.075</parameter>
    <parameter name="Minimum feature height">100000.0</parameter>
    <parameter name="Intensity threshold for correlation">100000.0</parameter>
    <parameter name="Min samples filter">
        <parameter name="Min samples in all">
            <abs>1</abs>
            <rel>0.0</rel>
        </parameter>
        <parameter name="Min samples in group">
            <abs>0</abs>
            <rel>0.0</rel>
        </parameter>
        <parameter name="Min %-intensity overlap">0.5</parameter>
        <parameter name="Exclude gap-filled features">true</parameter>
    </parameter>
    <parameter name="Feature shape correlation" selected="true">
        <parameter name="Min data points">5</parameter>
        <parameter name="Min data points on edge">2</parameter>
        <parameter name="Measure">PEARSON</parameter>
        <parameter name="Min feature shape correlation">0.65</parameter>
        <parameter name="Min total correlation" selected="false">0.5</parameter>
    </parameter>
    <parameter name="Feature height correlation" selected="true">
        <parameter name="Minimum samples">2</parameter>
        <parameter name="Measure">PEARSON</parameter>
        <parameter name="Min correlation">0.65</parameter>
    </parameter>
    <parameter name="Suffix (or auto)" selected="true">metaCor</parameter>
</batchstep>
<batchstep method="io.github.mzmine.modules.dataprocessing.id_ion_identity_networking.ionidnetworking.IonNetworkingModule"
      parameter_version="1">
    <parameter name="Feature lists" type="BATCH_LAST_FEATURELISTS"/>
    <parameter name="m/z tolerance (intra-sample)">
        <absolutetolerance>0.0</absolutetolerance>
        <ppmtolerance>4.0</ppmtolerance>
    </parameter>
    <parameter name="Check">ALL FEATURES</parameter>
    <parameter name="Min height">50.0</parameter>
    <parameter name="Ion identity library">
        <parameter name="MS mode">POSITIVE</parameter>
        <parameter name="Maximum charge">2</parameter>
        <parameter name="Maximum molecules/cluster">2</parameter>
        <parameter name="Adducts">
            <adduct_type selected="false">
                <subpart charge="-1" mass_difference="-1.007276" mol_formula="H" name="H" type="ADDUCT"/>
            </adduct_type>
            <adduct_type selected="false">
                <subpart charge="1" mass_difference="-5.4858E-4" mol_formula="" name="" type="ADDUCT"/>
            </adduct_type>
            <adduct_type selected="true">
                <subpart charge="1" mass_difference="1.007276" mol_formula="H" name="H" type="ADDUCT"/>
            </adduct_type>
            <adduct_type selected="true">
                <subpart charge="1" mass_difference="22.989218" mol_formula="Na" name="Na" type="ADDUCT"/>
            </adduct_type>
            <adduct_type selected="false">
                <subpart charge="1" mass_difference="38.963158" mol_formula="K" name="K" type="ADDUCT"/>
            </adduct_type>
            <adduct_type selected="true">
                <subpart charge="1" mass_difference="18.033823" mol_formula="H4N" name="NH4" type="ADDUCT"/>
            </adduct_type>
            <adduct_type selected="true">
                <subpart charge="1" mass_difference="1.007276" mol_formula="H" name="H" type="ADDUCT"/>
                <subpart charge="1" mass_difference="1.007276" mol_formula="H" name="H" type="ADDUCT"/>
            </adduct_type>
            <adduct_type selected="false">
                <subpart charge="2" mass_difference="39.96149382" mol_formula="Ca" name="Ca" type="ADDUCT"/>
            </adduct_type>
            <adduct_type selected="false">
                <subpart charge="2" mass_difference="55.93384" mol_formula="Fe" name="Fe" type="ADDUCT"/>
            </adduct_type>
            <adduct_type selected="false">
                <subpart charge="2" mass_difference="47.96953482" mol_formula="Mg" name="Mg" type="ADDUCT"/>
            </adduct_type>
            <adduct_type selected="true">
                <subpart charge="1" mass_difference="1.007276" mol_formula="H" name="H" type="ADDUCT"/>
                <subpart charge="1" mass_difference="22.989218" mol_formula="Na" name="Na" type="ADDUCT"/>
            </adduct_type>
            <adduct_type selected="true">
                <subpart charge="1" mass_difference="1.007276" mol_formula="H" name="H" type="ADDUCT"/>
                <subpart charge="1" mass_difference="18.033823" mol_formula="H4N" name="NH4" type="ADDUCT"/>
            </adduct_type>
            <adduct_type selected="false">
                <subpart charge="1" mass_difference="1.007276" mol_formula="H" name="H" type="ADDUCT"/>
                <subpart charge="1" mass_difference="38.963158" mol_formula="K" name="K" type="ADDUCT"/>
            </adduct_type>
            <adduct_type selected="true">
                <subpart charge="-1" mass_difference="-1.007276" mol_formula="H" name="H" type="ADDUCT"/>
                <subpart charge="1" mass_difference="22.989218" mol_formula="Na" name="Na" type="ADDUCT"/>
                <subpart charge="1" mass_difference="22.989218" mol_formula="Na" name="Na" type="ADDUCT"/>
            </adduct_type>
            <adduct_type selected="false">
                <subpart charge="2" mass_difference="39.96149382" mol_formula="Ca" name="Ca" type="ADDUCT"/>
                <subpart charge="-1" mass_difference="-1.007276" mol_formula="H" name="H" type="ADDUCT"/>
            </adduct_type>
            <adduct_type selected="false">
```

```
            <subpart charge="2" mass_difference="55.93384" mol_formula="Fe" name="Fe" type="ADDUCT"/>
            <subpart charge="-1" mass_difference="-1.007276" mol_formula="H" name="H" type="ADDUCT"/>
          </adduct_type>
          <adduct_type selected="false">
            <subpart charge="-1" mass_difference="-1.007276" mol_formula="H" name="H" type="ADDUCT"/>
            <subpart charge="2" mass_difference="47.96953482" mol_formula="Mg" name="Mg" type="ADDUCT"/>
          </adduct_type>
          <modification_type selected="true">
            <subpart charge="0" mass_difference="-15.023475" mol_formula="CH3" name="CH3" type="NEUTRAL_LOSS"/>
          </modification_type>
          <modification_type selected="true">
            <subpart charge="0" mass_difference="-17.026549" mol_formula="H3N" name="NH3" type="NEUTRAL_LOSS"/>
          </modification_type>
          <modification_type selected="true">
            <subpart charge="0" mass_difference="-18.010565" mol_formula="H2O" name="H2O" type="NEUTRAL_LOSS"/>
          </modification_type>
          <modification_type selected="true">
            <subpart charge="0" mass_difference="-18.010565" mol_formula="H2O" name="H2O" type="NEUTRAL_LOSS"/>
            <subpart charge="0" mass_difference="-18.010565" mol_formula="H2O" name="H2O" type="NEUTRAL_LOSS"/>
          </modification_type>
          <modification_type selected="true">
            <subpart charge="0" mass_difference="-146.05791" mol_formula="C6H10O4" name="C6H10O4" type="NEUTRAL_LOSS"/>
          </modification_type>
          <modification_type selected="true">
            <subpart charge="0" mass_difference="-162.052825" mol_formula="C6H10O5" name="C6H10O5" type="NEUTRAL_LOSS"/>
          </modification_type>
          <modification_type selected="true">
            <subpart charge="0" mass_difference="41.026549" mol_formula="C2H3N" name="C2H3N" type="CLUSTER"/>
          </modification_type>
        </parameter>
      </parameter>
      <parameter name="Annotation refinement" selected="true">
        <parameter name="Minimum size" selected="true">2</parameter>
        <parameter name="Delete small networks without major ion">true</parameter>
        <parameter name="Delete smaller networks: Link threshold" selected="true">3</parameter>
        <parameter name="Delete networks without monomer">true</parameter>
        <parameter name="Delete rows witout ion id">false</parameter>
      </parameter>
    </batchstep>
    <batchstep method="io.github.mzmine.modules.dataprocessing.id_ion_identity_networking.addionannotations.AddIonNetworkingModule"
          parameter_version="1">
      <parameter name="Feature lists" type="BATCH_LAST_FEATURELISTS"/>
      <parameter name="m/z tolerance (intra-sample)">
        <absolutetolerance>0.0</absolutetolerance>
        <ppmtolerance>12.0</ppmtolerance>
      </parameter>
      <parameter name="Min height">50.0</parameter>
      <parameter name="Annotation refinement" selected="true">
        <parameter name="Minimum size" selected="true">2</parameter>
        <parameter name="Delete small networks without major ion">true</parameter>
        <parameter name="Delete smaller networks: Link threshold" selected="true">3</parameter>
        <parameter name="Delete networks without monomer">true</parameter>
        <parameter name="Delete rows witout ion id">false</parameter>
      </parameter>
      <parameter name="Ion identity library">
        <parameter name="MS mode">POSITIVE</parameter>
        <parameter name="Maximum charge">2</parameter>
        <parameter name="Maximum molecules/cluster">2</parameter>
        <parameter name="Adducts">
          <adduct_type selected="true">
            <subpart charge="-1" mass_difference="-1.007276" mol_formula="H" name="H" type="ADDUCT"/>
          </adduct_type>
          <adduct_type selected="true">
            <subpart charge="1" mass_difference="-5.4858E-4" mol_formula="" name="" type="ADDUCT"/>
          </adduct_type>
          <adduct_type selected="true">
            <subpart charge="1" mass_difference="1.007276" mol_formula="H" name="H" type="ADDUCT"/>
          </adduct_type>
          <adduct_type selected="true">
            <subpart charge="1" mass_difference="22.989218" mol_formula="Na" name="Na" type="ADDUCT"/>
          </adduct_type>
          <adduct_type selected="true">
            <subpart charge="1" mass_difference="38.963158" mol_formula="K" name="K" type="ADDUCT"/>
          </adduct_type>
          <adduct_type selected="true">
            <subpart charge="1" mass_difference="18.033823" mol_formula="H4N" name="NH4" type="ADDUCT"/>
          </adduct_type>
          <adduct_type selected="true">
            <subpart charge="1" mass_difference="1.007276" mol_formula="H" name="H" type="ADDUCT"/>
            <subpart charge="1" mass_difference="1.007276" mol_formula="H" name="H" type="ADDUCT"/>
          </adduct_type>
          <adduct_type selected="true">
            <subpart charge="2" mass_difference="39.96149382" mol_formula="Ca" name="Ca" type="ADDUCT"/>
          </adduct_type>
          <adduct_type selected="true">
            <subpart charge="2" mass_difference="55.93384" mol_formula="Fe" name="Fe" type="ADDUCT"/>
          </adduct_type>
          <adduct_type selected="true">
            <subpart charge="2" mass_difference="47.96953482" mol_formula="Mg" name="Mg" type="ADDUCT"/>
          </adduct_type>
          <adduct_type selected="true">
            <subpart charge="1" mass_difference="1.007276" mol_formula="H" name="H" type="ADDUCT"/>
            <subpart charge="1" mass_difference="22.989218" mol_formula="Na" name="Na" type="ADDUCT"/>
          </adduct_type>
          <adduct_type selected="true">
            <subpart charge="1" mass_difference="1.007276" mol_formula="H" name="H" type="ADDUCT"/>
            <subpart charge="1" mass_difference="18.033823" mol_formula="H4N" name="NH4" type="ADDUCT"/>
          </adduct_type>
          <adduct_type selected="true">
```

```
            <subpart charge="1" mass_difference="1.007276" mol_formula="H" name="H" type="ADDUCT"/>
            <subpart charge="1" mass_difference="38.963158" mol_formula="K" name="K" type="ADDUCT"/>
        </adduct_type>
        <adduct_type selected="true">
            <subpart charge="-1" mass_difference="-1.007276" mol_formula="H" name="H" type="ADDUCT"/>
            <subpart charge="1" mass_difference="22.989218" mol_formula="Na" name="Na" type="ADDUCT"/>
            <subpart charge="1" mass_difference="22.989218" mol_formula="Na" name="Na" type="ADDUCT"/>
        </adduct_type>
        <adduct_type selected="true">
            <subpart charge="2" mass_difference="39.96149382" mol_formula="Ca" name="Ca" type="ADDUCT"/>
            <subpart charge="-1" mass_difference="-1.007276" mol_formula="H" name="H" type="ADDUCT"/>
        </adduct_type>
        <adduct_type selected="true">
            <subpart charge="2" mass_difference="55.93384" mol_formula="Fe" name="Fe" type="ADDUCT"/>
            <subpart charge="-1" mass_difference="-1.007276" mol_formula="H" name="H" type="ADDUCT"/>
        </adduct_type>
        <adduct_type selected="true">
            <subpart charge="-1" mass_difference="-1.007276" mol_formula="H" name="H" type="ADDUCT"/>
            <subpart charge="2" mass_difference="47.96953482" mol_formula="Mg" name="Mg" type="ADDUCT"/>
        </adduct_type>
      </parameter>
    </parameter>
</batchstep>
<batchstep method="io.github.mzmine.modules.dataprocessing.id_ion_identity_networking.addionannotations.AddIonNetworkingModule"
      parameter_version="1">
    <parameter name="Feature lists" type="BATCH_LAST_FEATURELISTS"/>
    <parameter name="m/z tolerance (intra-sample)">
        <absolutetolerance>0.0</absolutetolerance>
        <ppmtolerance>12.0</ppmtolerance>
    </parameter>
    <parameter name="Min height">50.0</parameter>
    <parameter name="Annotation refinement" selected="true">
        <parameter name="Minimum size" selected="false">2</parameter>
        <parameter name="Delete small networks without major ion">true</parameter>
        <parameter name="Delete smaller networks: Link threshold" selected="true">3</parameter>
        <parameter name="Delete networks without monomer">true</parameter>
        <parameter name="Delete rows witout ion id">false</parameter>
    </parameter>
    <parameter name="Ion identity library">
        <parameter name="MS mode">POSITIVE</parameter>
        <parameter name="Maximum charge">2</parameter>
        <parameter name="Maximum molecules/cluster">2</parameter>
        <parameter name="Adducts">
            <adduct_type selected="false">
                <subpart charge="-1" mass_difference="-1.007276" mol_formula="H" name="H" type="ADDUCT"/>
            </adduct_type>
            <adduct_type selected="false">
                <subpart charge="1" mass_difference="-5.4858E-4" mol_formula="" name="" type="ADDUCT"/>
            </adduct_type>
            <adduct_type selected="true">
                <subpart charge="1" mass_difference="1.007276" mol_formula="H" name="H" type="ADDUCT"/>
            </adduct_type>
            <adduct_type selected="true">
                <subpart charge="1" mass_difference="22.989218" mol_formula="Na" name="Na" type="ADDUCT"/>
            </adduct_type>
            <adduct_type selected="true">
                <subpart charge="1" mass_difference="38.963158" mol_formula="K" name="K" type="ADDUCT"/>
            </adduct_type>
            <adduct_type selected="true">
                <subpart charge="1" mass_difference="18.033823" mol_formula="H4N" name="NH4" type="ADDUCT"/>
            </adduct_type>
            <adduct_type selected="true">
                <subpart charge="1" mass_difference="1.007276" mol_formula="H" name="H" type="ADDUCT"/>
                <subpart charge="1" mass_difference="1.007276" mol_formula="H" name="H" type="ADDUCT"/>
            </adduct_type>
            <adduct_type selected="false">
                <subpart charge="2" mass_difference="39.96149382" mol_formula="Ca" name="Ca" type="ADDUCT"/>
            </adduct_type>
            <adduct_type selected="false">
                <subpart charge="2" mass_difference="55.93384" mol_formula="Fe" name="Fe" type="ADDUCT"/>
            </adduct_type>
            <adduct_type selected="false">
                <subpart charge="2" mass_difference="47.96953482" mol_formula="Mg" name="Mg" type="ADDUCT"/>
            </adduct_type>
            <adduct_type selected="false">
                <subpart charge="1" mass_difference="1.007276" mol_formula="H" name="H" type="ADDUCT"/>
                <subpart charge="1" mass_difference="22.989218" mol_formula="Na" name="Na" type="ADDUCT"/>
            </adduct_type>
            <adduct_type selected="false">
                <subpart charge="1" mass_difference="1.007276" mol_formula="H" name="H" type="ADDUCT"/>
                <subpart charge="1" mass_difference="18.033823" mol_formula="H4N" name="NH4" type="ADDUCT"/>
            </adduct_type>
            <adduct_type selected="false">
                <subpart charge="1" mass_difference="1.007276" mol_formula="H" name="H" type="ADDUCT"/>
                <subpart charge="1" mass_difference="38.963158" mol_formula="K" name="K" type="ADDUCT"/>
            </adduct_type>
            <adduct_type selected="true">
                <subpart charge="-1" mass_difference="-1.007276" mol_formula="H" name="H" type="ADDUCT"/>
                <subpart charge="1" mass_difference="22.989218" mol_formula="Na" name="Na" type="ADDUCT"/>
                <subpart charge="1" mass_difference="22.989218" mol_formula="Na" name="Na" type="ADDUCT"/>
            </adduct_type>
            <adduct_type selected="false">
                <subpart charge="2" mass_difference="39.96149382" mol_formula="Ca" name="Ca" type="ADDUCT"/>
                <subpart charge="-1" mass_difference="-1.007276" mol_formula="H" name="H" type="ADDUCT"/>
            </adduct_type>
            <adduct_type selected="false">
                <subpart charge="2" mass_difference="55.93384" mol_formula="Fe" name="Fe" type="ADDUCT"/>
                <subpart charge="-1" mass_difference="-1.007276" mol_formula="H" name="H" type="ADDUCT"/>
```

```
</adduct_type>
<adduct_type selected="false">
    <subpart charge="-1" mass_difference="-1.007276" mol_formula="H" name="H" type="ADDUCT"/>
    <subpart charge="2" mass_difference="47.96953482" mol_formula="Mg" name="Mg" type="ADDUCT"/>
</adduct_type>
<modification_type selected="true">
    <subpart charge="0" mass_difference="-15.0109" mol_formula="HN" name="NH" type="NEUTRAL_LOSS"/>
</modification_type>
<modification_type selected="true">
    <subpart charge="0" mass_difference="-15.023475" mol_formula="CH3" name="CH3" type="NEUTRAL_LOSS"/>
</modification_type>
<modification_type selected="true">
    <subpart charge="0" mass_difference="-17.026549" mol_formula="H3N" name="NH3" type="NEUTRAL_LOSS"/>
</modification_type>
<modification_type selected="true">
    <subpart charge="0" mass_difference="-18.010565" mol_formula="H2O" name="H2O" type="NEUTRAL_LOSS"/>
</modification_type>
<modification_type selected="true">
    <subpart charge="0" mass_difference="-18.010565" mol_formula="H2O" name="H2O" type="NEUTRAL_LOSS"/>
    <subpart charge="0" mass_difference="-18.010565" mol_formula="H2O" name="H2O" type="NEUTRAL_LOSS"/>
</modification_type>
<modification_type selected="true">
    <subpart charge="0" mass_difference="-18.010565" mol_formula="H2O" name="H2O" type="NEUTRAL_LOSS"/>
    <subpart charge="0" mass_difference="-18.010565" mol_formula="H2O" name="H2O" type="NEUTRAL_LOSS"/>
    <subpart charge="0" mass_difference="-18.010565" mol_formula="H2O" name="H2O" type="NEUTRAL_LOSS"/>
</modification_type>
<modification_type selected="true">
    <subpart charge="0" mass_difference="-18.010565" mol_formula="H2O" name="H2O" type="NEUTRAL_LOSS"/>
    <subpart charge="0" mass_difference="-18.010565" mol_formula="H2O" name="H2O" type="NEUTRAL_LOSS"/>
    <subpart charge="0" mass_difference="-18.010565" mol_formula="H2O" name="H2O" type="NEUTRAL_LOSS"/>
    <subpart charge="0" mass_difference="-18.010565" mol_formula="H2O" name="H2O" type="NEUTRAL_LOSS"/>
</modification_type>
<modification_type selected="true">
    <subpart charge="0" mass_difference="-18.010565" mol_formula="H2O" name="H2O" type="NEUTRAL_LOSS"/>
    <subpart charge="0" mass_difference="-18.010565" mol_formula="H2O" name="H2O" type="NEUTRAL_LOSS"/>
    <subpart charge="0" mass_difference="-18.010565" mol_formula="H2O" name="H2O" type="NEUTRAL_LOSS"/>
    <subpart charge="0" mass_difference="-18.010565" mol_formula="H2O" name="H2O" type="NEUTRAL_LOSS"/>
    <subpart charge="0" mass_difference="-18.010565" mol_formula="H2O" name="H2O" type="NEUTRAL_LOSS"/>
</modification_type>
<modification_type selected="true">
    <subpart charge="0" mass_difference="-27.0109" mol_formula="CHN" name="HCN" type="NEUTRAL_LOSS"/>
</modification_type>
<modification_type selected="true">
    <subpart charge="0" mass_difference="-27.994915" mol_formula="CO" name="CO" type="NEUTRAL_LOSS"/>
</modification_type>
<modification_type selected="true">
    <subpart charge="0" mass_difference="-27.994915" mol_formula="CO" name="CO" type="NEUTRAL_LOSS"/>
    <subpart charge="0" mass_difference="-27.994915" mol_formula="CO" name="CO" type="NEUTRAL_LOSS"/>
</modification_type>
<modification_type selected="true">
    <subpart charge="0" mass_difference="-27.994915" mol_formula="CO" name="CO" type="NEUTRAL_LOSS"/>
    <subpart charge="0" mass_difference="-27.994915" mol_formula="CO" name="CO" type="NEUTRAL_LOSS"/>
    <subpart charge="0" mass_difference="-27.994915" mol_formula="CO" name="CO" type="NEUTRAL_LOSS"/>
</modification_type>
<modification_type selected="true">
    <subpart charge="0" mass_difference="-14.01565" mol_formula="CH2" name="CH2" type="NEUTRAL_LOSS"/>
    <subpart charge="0" mass_difference="-14.01565" mol_formula="CH2" name="CH2" type="NEUTRAL_LOSS"/>
</modification_type>
<modification_type selected="true">
    <subpart charge="0" mass_difference="-14.01565" mol_formula="CH2" name="CH2" type="NEUTRAL_LOSS"/>
    <subpart charge="0" mass_difference="-15.02348" mol_formula="CH3" name="CH3" type="NEUTRAL_LOSS"/>
</modification_type>
<modification_type selected="true">
    <subpart charge="0" mass_difference="-30.01057" mol_formula="CH2O" name="CH2O" type="NEUTRAL_LOSS"/>
</modification_type>
<modification_type selected="true">
    <subpart charge="0" mass_difference="-32.02621" mol_formula="CH4O" name="CH4O" type="NEUTRAL_LOSS"/>
</modification_type>
<modification_type selected="true">
    <subpart charge="0" mass_difference="-42.010565" mol_formula="C2H2O" name="C2H2O" type="NEUTRAL_LOSS"/>
</modification_type>
<modification_type selected="true">
    <subpart charge="0" mass_difference="-43.00581" mol_formula="CHON" name="CHON" type="NEUTRAL_LOSS"/>
</modification_type>
<modification_type selected="true">
    <subpart charge="0" mass_difference="-43.989829" mol_formula="CO2" name="CO2" type="NEUTRAL_LOSS"/>
</modification_type>
<modification_type selected="true">
    <subpart charge="0" mass_difference="-43.989829" mol_formula="CO2" name="CO2" type="NEUTRAL_LOSS"/>
    <subpart charge="0" mass_difference="-43.989829" mol_formula="CO2" name="CO2" type="NEUTRAL_LOSS"/>
</modification_type>
<modification_type selected="true">
    <subpart charge="0" mass_difference="-27.994915" mol_formula="CO" name="CO" type="NEUTRAL_LOSS"/>
    <subpart charge="0" mass_difference="-18.010565" mol_formula="H2O" name="H2O" type="NEUTRAL_LOSS"/>
</modification_type>
<modification_type selected="true">
    <subpart charge="0" mass_difference="-46.00548" mol_formula="CH2O2" name="CH2O2" type="NEUTRAL_LOSS"/>
</modification_type>
<modification_type selected="true">
    <subpart charge="0" mass_difference="-46.04187" mol_formula="C2H6O" name="C2H6O" type="NEUTRAL_LOSS"/>
</modification_type>
<modification_type selected="true">
    <subpart charge="0" mass_difference="-60.02113" mol_formula="C2H4O2" name="C2H4O2" type="NEUTRAL_LOSS"/>
</modification_type>
<modification_type selected="true">
    <subpart charge="0" mass_difference="-66.0317" mol_formula="CH6O3" name="CH6O3" type="NEUTRAL_LOSS"/>
</modification_type>
<modification_type selected="true">
    <subpart charge="0" mass_difference="-30.01057" mol_formula="CH2O" name="CH2O" type="NEUTRAL_LOSS"/>
```

```
    <subpart charge="0" mass_difference="-43.989829" mol_formula="CO2" name="CO2" type="NEUTRAL_LOSS"/>
</modification_type>
<modification_type selected="true">
    <subpart charge="0" mass_difference="-14.01565" mol_formula="CH2" name="CH2" type="NEUTRAL_LOSS"/>
    <subpart charge="0" mass_difference="-46.005479" mol_formula="CH2O2" name="CH2O2" type="NEUTRAL_LOSS"/>
    <subpart charge="0" mass_difference="-15.02348" mol_formula="CH3" name="CH3" type="NEUTRAL_LOSS"/>
</modification_type>
<modification_type selected="true">
    <subpart charge="0" mass_difference="-87.06841" mol_formula="C4H9NO" name="C4H9NO" type="NEUTRAL_LOSS"/>
</modification_type>
<modification_type selected="true">
    <subpart charge="0" mass_difference="-88.01604" mol_formula="C3H4O3" name="C3H4O3" type="NEUTRAL_LOSS"/>
</modification_type>
<modification_type selected="true">
    <subpart charge="0" mass_difference="-88.05243" mol_formula="C4H8O2" name="C4H8O2" type="NEUTRAL_LOSS"/>
</modification_type>
<modification_type selected="true">
    <subpart charge="0" mass_difference="-89.04768" mol_formula="C3H7NO2" name="C3H7NO2" type="NEUTRAL_LOSS"/>
</modification_type>
<modification_type selected="true">
    <subpart charge="0" mass_difference="-90.031695" mol_formula="C3H6O3" name="C3H6O3" type="NEUTRAL_LOSS"/>
</modification_type>
<modification_type selected="true">
    <subpart charge="0" mass_difference="-97.96738" mol_formula="H2O4S" name="H2O4S" type="NEUTRAL_LOSS"/>
</modification_type>
<modification_type selected="true">
    <subpart charge="0" mass_difference="-97.9769" mol_formula="H3O4P" name="H3O4P" type="NEUTRAL_LOSS"/>
</modification_type>
<modification_type selected="true">
    <subpart charge="0" mass_difference="-102.06808" mol_formula="C5H10O2" name="C5H10O2" type="NEUTRAL_LOSS"/>
</modification_type>
<modification_type selected="true">
    <subpart charge="0" mass_difference="-104.01096" mol_formula="C3H4O4" name="C3H4O4" type="NEUTRAL_LOSS"/>
</modification_type>
<modification_type selected="true">
    <subpart charge="0" mass_difference="-43.989829" mol_formula="CO2" name="CO2" type="NEUTRAL_LOSS"/>
    <subpart charge="0" mass_difference="-43.989829" mol_formula="CO2" name="CO2" type="NEUTRAL_LOSS"/>
    <subpart charge="0" mass_difference="-18.010565" mol_formula="H2O" name="H2O" type="NEUTRAL_LOSS"/>
</modification_type>
<modification_type selected="true">
    <subpart charge="0" mass_difference="-108.04226" mol_formula="C3H8O4" name="C3H8O4" type="NEUTRAL_LOSS"/>
</modification_type>
<modification_type selected="true">
    <subpart charge="0" mass_difference="-115.06333" mol_formula="C5H9NO2" name="C5H9NO2" type="NEUTRAL_LOSS"/>
</modification_type>
<modification_type selected="true">
    <subpart charge="0" mass_difference="-15.023475" mol_formula="CH3" name="CH3" type="NEUTRAL_LOSS"/>
    <subpart charge="0" mass_difference="-15.023475" mol_formula="CH3" name="CH3" type="NEUTRAL_LOSS"/>
    <subpart charge="0" mass_difference="-43.989829" mol_formula="CO2" name="CO2" type="NEUTRAL_LOSS"/>
    <subpart charge="0" mass_difference="-43.989829" mol_formula="CO2" name="CO2" type="NEUTRAL_LOSS"/>
</modification_type>
<modification_type selected="true">
    <subpart charge="0" mass_difference="-120.04226" mol_formula="C4H8O4" name="C4H8O4" type="NEUTRAL_LOSS"/>
</modification_type>
<modification_type selected="true">
    <subpart charge="0" mass_difference="-43.989829" mol_formula="CO2" name="CO2" type="NEUTRAL_LOSS"/>
    <subpart charge="0" mass_difference="-43.989829" mol_formula="CO2" name="CO2" type="NEUTRAL_LOSS"/>
    <subpart charge="0" mass_difference="-18.010565" mol_formula="H2O" name="H2O" type="NEUTRAL_LOSS"/>
    <subpart charge="0" mass_difference="-18.010565" mol_formula="H2O" name="H2O" type="NEUTRAL_LOSS"/>
</modification_type>
<modification_type selected="true">
    <subpart charge="0" mass_difference="-126.03169" mol_formula="C6H6O3" name="C6H6O3" type="NEUTRAL_LOSS"/>
</modification_type>
<modification_type selected="true">
    <subpart charge="0" mass_difference="-132.04226" mol_formula="C5H8O4" name="C5H8O4" type="NEUTRAL_LOSS"/>
</modification_type>
<modification_type selected="true">
    <subpart charge="0" mass_difference="-136.05243" mol_formula="C8H8O2" name="C8H8O2" type="NEUTRAL_LOSS"/>
</modification_type>
<modification_type selected="true">
    <subpart charge="0" mass_difference="-144.04226" mol_formula="C6H8O4" name="C6H8O4" type="NEUTRAL_LOSS"/>
</modification_type>
<modification_type selected="true">
    <subpart charge="0" mass_difference="-146.03678" mol_formula="C9H6O2" name="C9H6O2" type="NEUTRAL_LOSS"/>
</modification_type>
<modification_type selected="true">
    <subpart charge="0" mass_difference="-146.05791" mol_formula="C6H10O4" name="C6H10O4" type="NEUTRAL_LOSS"/>
</modification_type>
<modification_type selected="true">
    <subpart charge="0" mass_difference="-146.05791" mol_formula="C6H10O4" name="C6H10O4" type="NEUTRAL_LOSS"/>
    <subpart charge="0" mass_difference="-146.05791" mol_formula="C6H10O4" name="C6H10O4" type="NEUTRAL_LOSS"/>
</modification_type>
<modification_type selected="true">
    <subpart charge="0" mass_difference="-148.05243" mol_formula="C9H8O2" name="C9H8O2" type="NEUTRAL_LOSS"/>
</modification_type>
<modification_type selected="true">
    <subpart charge="0" mass_difference="-152.01096" mol_formula="C7H4O4" name="C7H4O4" type="NEUTRAL_LOSS"/>
</modification_type>
<modification_type selected="true">
    <subpart charge="0" mass_difference="-152.04734" mol_formula="C8H8O3" name="C8H8O3" type="NEUTRAL_LOSS"/>
</modification_type>
<modification_type selected="true">
    <subpart charge="0" mass_difference="-162.03169" mol_formula="C9H6O3" name="C9H6O3" type="NEUTRAL_LOSS"/>
</modification_type>
<modification_type selected="true">
    <subpart charge="0" mass_difference="-162.05282" mol_formula="C6H10O5" name="C6H10O5" type="NEUTRAL_LOSS"/>
</modification_type>
<modification_type selected="true">
    <subpart charge="0" mass_difference="-162.05282" mol_formula="C6H10O5" name="C6H10O5" type="NEUTRAL_LOSS"/>
```

```
            <subpart charge="0" mass_difference="-162.05282" mol_formula="C6H10O5" name="C6H10O5" type="NEUTRAL_LOSS"/>
        </modification_type>
        <modification_type selected="true">
            <subpart charge="0" mass_difference="-162.05282" mol_formula="C6H10O5" name="C6H10O5" type="NEUTRAL_LOSS"/>
            <subpart charge="0" mass_difference="-162.05282" mol_formula="C6H10O5" name="C6H10O5" type="NEUTRAL_LOSS"/>
            <subpart charge="0" mass_difference="-162.05282" mol_formula="C6H10O5" name="C6H10O5" type="NEUTRAL_LOSS"/>
        </modification_type>
        <modification_type selected="true">
            <subpart charge="0" mass_difference="-168.04226" mol_formula="C8H8O4" name="C8H8O4" type="NEUTRAL_LOSS"/>
        </modification_type>
        <modification_type selected="true">
            <subpart charge="0" mass_difference="-170.05791" mol_formula="C8H10O4" name="C8H10O4" type="NEUTRAL_LOSS"/>
        </modification_type>
        <modification_type selected="true">
            <subpart charge="0" mass_difference="-176.03209" mol_formula="C6H8O6" name="C6H8O6" type="NEUTRAL_LOSS"/>
        </modification_type>
        <modification_type selected="true">
            <subpart charge="0" mass_difference="-176.04734" mol_formula="C10H8O3" name="C10H8O3" type="NEUTRAL_LOSS"/>
        </modification_type>
        <modification_type selected="true">
            <subpart charge="0" mass_difference="-179.07937" mol_formula="C6H13NO5" name="C6H13NO5" type="NEUTRAL_LOSS"/>
        </modification_type>
        <modification_type selected="true">
            <subpart charge="0" mass_difference="-180.06339" mol_formula="C6H12O6" name="C6H12O6" type="NEUTRAL_LOSS"/>
        </modification_type>
        <modification_type selected="true">
            <subpart charge="0" mass_difference="-192.06339" mol_formula="C7H12O6" name="C7H12O6" type="NEUTRAL_LOSS"/>
        </modification_type>
        <modification_type selected="true">
            <subpart charge="0" mass_difference="-197.08994" mol_formula="C6H12O6" name="C6H12O6" type="NEUTRAL_LOSS"/>
        </modification_type>
        <modification_type selected="true">
            <subpart charge="0" mass_difference="-198.07395" mol_formula="C6H14O7" name="C6H14O7" type="NEUTRAL_LOSS"/>
        </modification_type>
        <modification_type selected="true">
            <subpart charge="0" mass_difference="-204.06339" mol_formula="C8H12O6" name="C8H12O6" type="NEUTRAL_LOSS"/>
        </modification_type>
        <modification_type selected="true">
            <subpart charge="0" mass_difference="-206.05791" mol_formula="C11H10O4" name="C11H10O4" type="NEUTRAL_LOSS"/>
        </modification_type>
        <modification_type selected="true">
            <subpart charge="0" mass_difference="-238.22967" mol_formula="C16H30O" name="C16H30O" type="NEUTRAL_LOSS"/>
        </modification_type>
        <modification_type selected="true">
            <subpart charge="0" mass_difference="-248.05322" mol_formula="C9H12O8" name="C9H12O8" type="NEUTRAL_LOSS"/>
        </modification_type>
        <modification_type selected="true">
            <subpart charge="0" mass_difference="-266.07904" mol_formula="C13H14O6" name="C13H14O6" type="NEUTRAL_LOSS"/>
        </modification_type>
        <modification_type selected="true">
            <subpart charge="0" mass_difference="-308.0896" mol_formula="C15H16O7" name="C15H16O7" type="NEUTRAL_LOSS"/>
        </modification_type>
        <modification_type selected="true">
            <subpart charge="0" mass_difference="-314.06378" mol_formula="C13H14O9" name="C13H14O9" type="NEUTRAL_LOSS"/>
        </modification_type>
        <modification_type selected="true">
            <subpart charge="0" mass_difference="-324.08452" mol_formula="C15H16O8" name="C15H16O8" type="NEUTRAL_LOSS"/>
        </modification_type>
        <modification_type selected="true">
            <subpart charge="0" mass_difference="-338.10017" mol_formula="C16H18O8" name="C16H18O8" type="NEUTRAL_LOSS"/>
        </modification_type>
        <modification_type selected="true">
            <subpart charge="0" mass_difference="-368.11073" mol_formula="C17H20O9" name="C17H20O9" type="NEUTRAL_LOSS"/>
        </modification_type>
        <modification_type selected="true">
            <subpart charge="0" mass_difference="15.99491462" mol_formula="O" name="O" type="CLUSTER"/>
        </modification_type>
        <modification_type selected="true">
            <subpart charge="0" mass_difference="45.05785" mol_formula="C2H7N" name="C2H7N" type="CLUSTER"/>
        </modification_type>
        <modification_type selected="true">
            <subpart charge="0" mass_difference="46.005479" mol_formula="CH2O2" name="CH2O2" type="CLUSTER"/>
        </modification_type>
        <modification_type selected="true">
            <subpart charge="0" mass_difference="60.021129" mol_formula="C2H4O2" name="C2H4O2" type="CLUSTER"/>
        </modification_type>
        <modification_type selected="true">
            <subpart charge="0" mass_difference="32.026215" mol_formula="CH4O" name="CH4O" type="CLUSTER"/>
        </modification_type>
        <modification_type selected="true">
            <subpart charge="0" mass_difference="41.026549" mol_formula="C2H3N" name="C2H3N" type="CLUSTER"/>
        </modification_type>
        <modification_type selected="true">
            <subpart charge="0" mass_difference="60.058064" mol_formula="C3H8O" name="C3H8O" type="CLUSTER"/>
        </modification_type>
    </parameter>
  </parameter>
</batchstep>
<batchstep method="io.github.mzmine.modules.dataprocessing.filter_rowsfilter.RowsFilterModule" parameter_version="2">
    <parameter name="Feature lists" type="BATCH_LAST_FEATURELISTS"/>
    <parameter name="Name suffix">filtered</parameter>
    <parameter name="Minimum aligned features (samples)" selected="false">
        <abs>1</abs>
        <rel>0.0</rel>
    </parameter>
    <parameter name="Minimum features in an isotope pattern" selected="false">2</parameter>
    <parameter name="Validate 13C isotope pattern" selected="false">
        <parameter name="m/z tolerance">
            <absolutetolerance>0.0</absolutetolerance>
```

```
            <ppmtolerance>12.0</ppmtolerance>
        </parameter>
        <parameter name="Max charge">1</parameter>
        <parameter name="Estimate minimum carbon">true</parameter>
        <parameter name="Remove if 13C">true</parameter>
        <parameter name="Exclude isotopes">H,C,N,O,S,P</parameter>
    </parameter>
    <parameter name="Remove redundant isotope rows">false</parameter>
    <parameter name="m/z" selected="false"/>
    <parameter name="Retention time" selected="false"/>
    <parameter name="features duration range" selected="false">
        <min>0.0</min>
        <max>3.0</max>
    </parameter>
    <parameter name="Chromatographic FWHM" selected="false">
        <min>0.0</min>
        <max>1.0</max>
    </parameter>
    <parameter name="Charge" selected="false">
        <min>1</min>
        <max>2</max>
    </parameter>
    <parameter name="Kendrick mass defect" selected="false">
        <parameter name="Kendrick mass defect">
            <min>0.0</min>
            <max>1.0</max>
        </parameter>
        <parameter name="Kendrick mass base"/>
        <parameter name="Shift">0.0</parameter>
        <parameter name="Charge">1</parameter>
        <parameter name="Divisor">1</parameter>
        <parameter name="Use Remainder of Kendrick mass">false</parameter>
    </parameter>
    <parameter name="Parameter">No parameters defined</parameter>
    <parameter name="Only identified?">false</parameter>
    <parameter name="Text in identity" selected="false"/>
    <parameter name="Text in comment" selected="false"/>
    <parameter name="Keep or remove rows">Keep rows that match all criteria</parameter>
    <parameter name="Feature with MS2 scan">true</parameter>
    <parameter name="Never remove feature with MS2">true</parameter>
    <parameter name="Reset the feature number ID">true</parameter>
    <parameter name="Mass defect" selected="false"/>
    <parameter name="Original feature list">PROCESS IN PLACE</parameter>
</batchstep>
<batchstep method="io.github.mzmine.modules.io.export_features_gnps.fbmn.GnpsFbmnExportAndSubmitModule" parameter_version="2">
    <parameter name="Feature lists" type="BATCH_LAST_FEATURELISTS"/>
    <parameter name="Filename">
        <current_file>data/interim/mzmine/lists/MYFILENAME_gnps</current_file>
    </parameter>
    <parameter name="Merge MS/MS (experimental)" selected="true">
        <parameter name="Select spectra to merge">across samples</parameter>
        <parameter name="m/z merge mode">weighted average (remove outliers)</parameter>
        <parameter name="intensity merge mode">sum intensities</parameter>
        <parameter name="Expected mass deviation">
            <absolutetolerance>0.0</absolutetolerance>
            <ppmtolerance>6.0</ppmtolerance>
        </parameter>
        <parameter name="Cosine threshold (%)">0.7</parameter>
        <parameter name="Signal count threshold (%)">0.2</parameter>
        <parameter name="Isolation window offset (m/z)">0.0</parameter>
        <parameter name="Isolation window width (m/z)">3.0</parameter>
    </parameter>
    <parameter name="Filter rows">MS2 OR ION IDENTITY</parameter>
    <parameter name="Feature intensity">Area</parameter>
    <parameter name="CSV export">ALL</parameter>
    <parameter name="Submit to GNPS" selected="false">
        <parameter name="Meta data file" selected="false"/>
        <parameter name="Export ion identity networks">true</parameter>
        <parameter name="Presets">HIGHRES</parameter>
        <parameter name="Job title"/>
        <parameter name="Email"/>
        <parameter name="Username"/>
        <parameter name="Password"/>
        <parameter name="Open website">true</parameter>
    </parameter>
    <parameter name="Open folder">false</parameter>
</batchstep>
<batchstep method="io.github.mzmine.modules.io.export_features_sirius.SiriusExportModule" parameter_version="1">
    <parameter name="Feature lists" type="BATCH_LAST_FEATURELISTS"/>
    <parameter name="Filename">
        <current_file>data/interim/mzmine/mgfs/MYFILENAME_sirius.mgf</current_file>
    </parameter>
    <parameter name="Merge MS/MS" selected="true">
        <parameter name="Select spectra to merge">across samples</parameter>
        <parameter name="m/z merge mode">weighted average (remove outliers)</parameter>
        <parameter name="intensity merge mode">sum intensities</parameter>
        <parameter name="Expected mass deviation">
            <absolutetolerance>0.0</absolutetolerance>
            <ppmtolerance>6.0</ppmtolerance>
        </parameter>
        <parameter name="Cosine threshold (%)">0.7</parameter>
        <parameter name="Signal count threshold (%)">0.2</parameter>
        <parameter name="Isolation window offset (m/z)">0.0</parameter>
        <parameter name="Isolation window width (m/z)">3.0</parameter>
    </parameter>
    <parameter name="m/z tolerance">
```

```
            <absolutetolerance>0.0</absolutetolerance>
            <ppmtolerance>6.0</ppmtolerance>
        </parameter>
        <parameter name="Only rows with annotation">false</parameter>
        <parameter name="Exclude multiple charge">false</parameter>
        <parameter name="Exclude multimers">false</parameter>
    </batchstep>
</batch>
}
```

3.2 MZmine template (negative)

```
<?xml version="1.0" encoding="UTF-8"?><batch mzmine_version="3.4.0">
    <batchstep method="io.github.mzmine.modules.io.import_rawdata_all.AllSpectralDataImportModule" parameter_version="1">
        <parameter name="File names">
            <file>MYFILEPATH</file>
        </parameter>
        <parameter name="Advanced import" selected="false">
            <parameter name="MS1 detector (Advanced)" selected="false" selected_item="Factor of lowest signal">
                <module name="Factor of lowest signal">
                    <parameter name="Noise factor">2.5</parameter>
                </module>
                <module name="Centroid">
                    <parameter name="Noise level">0.0</parameter>
                    <parameter name="Detect isotope signals below noise level" selected="true">
                        <parameter name="Chemical elements">H,C,N,O,S,P</parameter>
                        <parameter name="m/z tolerance">
                            <absolutetolerance>0.0</absolutetolerance>
                            <ppmtolerance>4.0</ppmtolerance>
                        </parameter>
                        <parameter name="Maximum charge of isotope m/z">2</parameter>
                    </parameter>
                </module>
                <module name="Exact mass">
                    <parameter name="Noise level"/>
                    <parameter name="Detect isotope signals below noise level" selected="true">
                        <parameter name="Chemical elements">H,C,N,O,S,P</parameter>
                        <parameter name="m/z tolerance">
                            <absolutetolerance>0.0</absolutetolerance>
                            <ppmtolerance>4.0</ppmtolerance>
                        </parameter>
                        <parameter name="Maximum charge of isotope m/z">2</parameter>
                    </parameter>
                </module>
                <module name="Local maxima">
                    <parameter name="Noise level"/>
                </module>
                <module name="Recursive threshold">
                    <parameter name="Noise level"/>
                    <parameter name="Min m/z peak width"/>
                    <parameter name="Max m/z peak width"/>
                </module>
                <module name="Wavelet transform">
                    <parameter name="Noise level"/>
                    <parameter name="Scale level"/>
                    <parameter name="Wavelet window size (%)"/>
                </module>
                <module name="Auto">
                    <parameter name="Noise level">10000.0</parameter>
                    <parameter name="Detect isotope signals below noise level" selected="true">
                        <parameter name="Chemical elements">H,C,N,O,S,P</parameter>
                        <parameter name="m/z tolerance">
                            <absolutetolerance>0.0</absolutetolerance>
                            <ppmtolerance>4.0</ppmtolerance>
                        </parameter>
                        <parameter name="Maximum charge of isotope m/z">2</parameter>
                    </parameter>
                </module>
            </parameter>
            <parameter name="MS2 detector (Advanced)" selected="false" selected_item="Factor of lowest signal">
                <module name="Factor of lowest signal">
                    <parameter name="Noise factor">2.5</parameter>
                </module>
                <module name="Centroid">
                    <parameter name="Noise level">0.0</parameter>
                    <parameter name="Detect isotope signals below noise level" selected="true">
                        <parameter name="Chemical elements">H,C,N,O,S,P</parameter>
                        <parameter name="m/z tolerance">
                            <absolutetolerance>0.0</absolutetolerance>
                            <ppmtolerance>4.0</ppmtolerance>
                        </parameter>
                        <parameter name="Maximum charge of isotope m/z">2</parameter>
                    </parameter>
                </module>
                <module name="Exact mass">
                    <parameter name="Noise level"/>
                    <parameter name="Detect isotope signals below noise level" selected="true">
                        <parameter name="Chemical elements">H,C,N,O,S,P</parameter>
                        <parameter name="m/z tolerance">
                            <absolutetolerance>0.0</absolutetolerance>
                            <ppmtolerance>4.0</ppmtolerance>
```

```
                </parameter>
                <parameter name="Maximum charge of isotope m/z">2</parameter>
            </parameter>
        </module>
        <module name="Local maxima">
            <parameter name="Noise level"/>
        </module>
        <module name="Recursive threshold">
            <parameter name="Noise level"/>
            <parameter name="Min m/z peak width"/>
            <parameter name="Max m/z peak width"/>
        </module>
        <module name="Wavelet transform">
            <parameter name="Noise level"/>
            <parameter name="Scale level"/>
            <parameter name="Wavelet window size (%)"/>
        </module>
        <module name="Auto">
            <parameter name="Noise level">0.0</parameter>
            <parameter name="Detect isotope signals below noise level" selected="true">
                <parameter name="Chemical elements">H,C,N,O,S,P</parameter>
                <parameter name="m/z tolerance">
                    <absolutetolerance>0.0</absolutetolerance>
                    <ppmtolerance>4.0</ppmtolerance>
                </parameter>
                <parameter name="Maximum charge of isotope m/z">2</parameter>
            </parameter>
        </module>
    </parameter>
    <parameter name="Denormalize fragment scans (traps)">false</parameter>
</parameter>
<parameter name="Spectral library files"/>
</batchstep>
<batchstep method="io.github.mzmine.modules.dataprocessing.featdet_massdetection.MassDetectionModule" parameter_version="1">
    <parameter name="Raw data files" type="BATCH_LAST_FILES"/>
    <parameter name="Scan filters" selected="true">
        <parameter name="Scan number"/>
        <parameter name="Base Filtering Integer"/>
        <parameter name="Retention time"/>
        <parameter name="Mobility"/>
        <parameter name="MS level filter" selected="MS2, level = 2">2</parameter>
        <parameter name="Scan definition"/>
        <parameter name="Polarity">Any</parameter>
        <parameter name="Spectrum type">ANY</parameter>
    </parameter>
    <parameter name="Scan types (IMS)">All scan types</parameter>
    <parameter name="Mass detector" selected_item="Auto">
        <module name="Factor of lowest signal">
            <parameter name="Noise factor">2.5</parameter>
        </module>
        <module name="Centroid">
            <parameter name="Noise level">0.0</parameter>
            <parameter name="Detect isotope signals below noise level" selected="true">
                <parameter name="Chemical elements">H,C,N,O,S,P</parameter>
                <parameter name="m/z tolerance">
                    <absolutetolerance>0.0</absolutetolerance>
                    <ppmtolerance>4.0</ppmtolerance>
                </parameter>
                <parameter name="Maximum charge of isotope m/z">2</parameter>
            </parameter>
        </module>
        <module name="Exact mass">
            <parameter name="Noise level"/>
            <parameter name="Detect isotope signals below noise level" selected="true">
                <parameter name="Chemical elements">H,C,N,O,S,P</parameter>
                <parameter name="m/z tolerance">
                    <absolutetolerance>0.0</absolutetolerance>
                    <ppmtolerance>4.0</ppmtolerance>
                </parameter>
                <parameter name="Maximum charge of isotope m/z">2</parameter>
            </parameter>
        </module>
        <module name="Local maxima">
            <parameter name="Noise level"/>
        </module>
        <module name="Recursive threshold">
            <parameter name="Noise level"/>
            <parameter name="Min m/z peak width"/>
            <parameter name="Max m/z peak width"/>
        </module>
        <module name="Wavelet transform">
            <parameter name="Noise level"/>
            <parameter name="Scale level"/>
            <parameter name="Wavelet window size (%)"/>
        </module>
        <module name="Auto">
            <parameter name="Noise level">10000.0</parameter>
            <parameter name="Detect isotope signals below noise level" selected="true">
                <parameter name="Chemical elements">H,C,N,O,S,P</parameter>
                <parameter name="m/z tolerance">
                    <absolutetolerance>0.0</absolutetolerance>
                    <ppmtolerance>4.0</ppmtolerance>
                </parameter>
                <parameter name="Maximum charge of isotope m/z">2</parameter>
            </parameter>
        </module>
    </parameter>
```

```
    <parameter name="Denormalize fragment scans (traps)">false</parameter>
    <parameter name="Output netCDF filename (optional)" selected="false"/>
</batchstep>
<batchstep method="io.github.mzmine.modules.dataprocessing.featdet_massdetection.MassDetectionModule" parameter_version="1">
    <parameter name="Raw data files" type="BATCH_LAST_FILES"/>
    <parameter name="Scan filters" selected="true">
        <parameter name="Scan number"/>
        <parameter name="Base Filtering Integer"/>
        <parameter name="Retention time"/>
        <parameter name="Mobility"/>
        <parameter name="MS level filter" selected="MS1, level = 1">1</parameter>
        <parameter name="Scan definition"/>
        <parameter name="Polarity">Any</parameter>
        <parameter name="Spectrum type">ANY</parameter>
    </parameter>
    <parameter name="Scan types (IMS)">All scan types</parameter>
    <parameter name="Mass detector" selected_item="Auto">
        <module name="Factor of lowest signal">
            <parameter name="Noise factor">2.5</parameter>
        </module>
        <module name="Centroid">
            <parameter name="Noise level">0.0</parameter>
            <parameter name="Detect isotope signals below noise level" selected="true">
                <parameter name="Chemical elements">H,C,N,O,S,P</parameter>
                <parameter name="m/z tolerance">
                    <absolutetolerance>0.0</absolutetolerance>
                    <ppmtolerance>4.0</ppmtolerance>
                </parameter>
                <parameter name="Maximum charge of isotope m/z">2</parameter>
            </parameter>
        </module>
        <module name="Exact mass">
            <parameter name="Noise level"/>
            <parameter name="Detect isotope signals below noise level" selected="true">
                <parameter name="Chemical elements">H,C,N,O,S,P</parameter>
                <parameter name="m/z tolerance">
                    <absolutetolerance>0.0</absolutetolerance>
                    <ppmtolerance>4.0</ppmtolerance>
                </parameter>
                <parameter name="Maximum charge of isotope m/z">2</parameter>
            </parameter>
        </module>
        <module name="Local maxima">
            <parameter name="Noise level"/>
        </module>
        <module name="Recursive threshold">
            <parameter name="Noise level"/>
            <parameter name="Min m/z peak width"/>
            <parameter name="Max m/z peak width"/>
        </module>
        <module name="Wavelet transform">
            <parameter name="Noise level"/>
            <parameter name="Scale level"/>
            <parameter name="Wavelet window size (%)"/>
        </module>
        <module name="Auto">
            <parameter name="Noise level">0.0</parameter>
            <parameter name="Detect isotope signals below noise level" selected="true">
                <parameter name="Chemical elements">H,C,N,O,S,P</parameter>
                <parameter name="m/z tolerance">
                    <absolutetolerance>0.0</absolutetolerance>
                    <ppmtolerance>4.0</ppmtolerance>
                </parameter>
                <parameter name="Maximum charge of isotope m/z">2</parameter>
            </parameter>
        </module>
    </parameter>
    <parameter name="Denormalize fragment scans (traps)">false</parameter>
    <parameter name="Output netCDF filename (optional)" selected="false"/>
</batchstep>
<batchstep method="io.github.mzmine.modules.dataprocessing.featdet_adapchromatogrambuilder.ModularADAPChromatogramBuilderModule"
      parameter_version="1">
    <parameter name="Raw data files" type="BATCH_LAST_FILES"/>
    <parameter name="Scan filters" selected="true">
        <parameter name="Scan number"/>
        <parameter name="Base Filtering Integer"/>
        <parameter name="Retention time">
            <min>0.0</min>
            <max>150.0</max>
        </parameter>
        <parameter name="Mobility"/>
        <parameter name="MS level filter" selected="MS1, level = 1">1</parameter>
        <parameter name="Scan definition"/>
        <parameter name="Polarity">Any</parameter>
        <parameter name="Spectrum type">ANY</parameter>
    </parameter>
    <parameter name="Minimum consecutive scans">4</parameter>
    <parameter name="Minimum intensity for consecutive scans">10000.0</parameter>
    <parameter name="Minimum absolute height">50000.0</parameter>
    <parameter name="m/z tolerance (scan-to-scan)">
        <absolutetolerance>0.0</absolutetolerance>
        <ppmtolerance>12.0</ppmtolerance>
    </parameter>
    <parameter name="Suffix">eics</parameter>
    <parameter name="Allow single scan chromatograms"/>
</batchstep>
<batchstep method="io.github.mzmine.modules.dataprocessing.featdet_smoothing.SmoothingModule" parameter_version="1">
```

```
    <parameter name="Feature lists" type="BATCH_LAST_FEATURELISTS"/>
    <parameter name="Smoothing algorithm" selected_item="Savitzky Golay">
        <module name="Savitzky Golay">
            <parameter name="Retention time smoothing" selected="true">5</parameter>
            <parameter name="Mobility smoothing" selected="false">5</parameter>
        </module>
        <module name="Loess smoothing">
            <parameter name="Retention time width (scans)" selected="false">5</parameter>
            <parameter name="Mobility width (scans)" selected="false">5</parameter>
        </module>
    </parameter>
    <parameter name="Original feature list">REMOVE</parameter>
    <parameter name="Suffix">sm</parameter>
</batchstep>
<batchstep method="io.github.mzmine.modules.dataprocessing.featdet_chromatogramdeconvolution.minimumsearch.MinimumSearchFeatureResolverModule"
       parameter_version="2">
    <parameter name="Feature lists" type="BATCH_LAST_FEATURELISTS"/>
    <parameter name="Suffix">r</parameter>
    <parameter name="Original feature list">REMOVE</parameter>
    <parameter name="MS/MS scan pairing" selected="true">
        <parameter name="Minimum relative feature height" selected="true">0.15</parameter>
        <parameter name="Minimum required signals" selected="true">1</parameter>
        <parameter name="MS1 to MS2 precursor tolerance (m/z)">
            <absolutetolerance>0.0</absolutetolerance>
            <ppmtolerance>12.0</ppmtolerance>
        </parameter>
        <parameter name="Retention time filter" selected="Use feature edges" unit="MINUTES">0.05</parameter>
        <parameter name="Limit by ion mobility edges">true</parameter>
        <parameter name="Merge MS/MS spectra (TIMS)">false</parameter>
        <parameter name="Minimum signal intensity (absolute, TIMS)" selected="false">6000.0</parameter>
        <parameter name="Minimum signal intensity (relative, TIMS)" selected="false">0.1</parameter>
    </parameter>
    <parameter name="Dimension">Retention time</parameter>
    <parameter name="Chromatographic threshold">0.9</parameter>
    <parameter name="Minimum search range RT/Mobility (absolute)">0.05</parameter>
    <parameter name="Minimum relative height">0.0</parameter>
    <parameter name="Minimum absolute height">10000.0</parameter>
    <parameter name="Min ratio of peak top/edge">1.8</parameter>
    <parameter name="Peak duration range (min/mobility)">
        <min>0.0</min>
        <max>1.5</max>
    </parameter>
    <parameter name="Minimum scans (data points)">5</parameter>
</batchstep>
<batchstep method="io.github.mzmine.modules.dataprocessing.filter_isotopegrouper.IsotopeGrouperModule" parameter_version="1">
    <parameter name="Feature lists" type="BATCH_LAST_FEATURELISTS"/>
    <parameter name="Name suffix">deiso</parameter>
    <parameter name="m/z tolerance (intra-sample)">
        <absolutetolerance>0.0</absolutetolerance>
        <ppmtolerance>4.0</ppmtolerance>
    </parameter>
    <parameter name="Retention time tolerance" unit="MINUTES">0.04</parameter>
    <parameter name="Mobility tolerance" selected="false">1.0</parameter>
    <parameter name="Monotonic shape">true</parameter>
    <parameter name="Maximum charge">2</parameter>
    <parameter name="Representative isotope">Most intense</parameter>
    <parameter name="Never remove feature with MS2">true</parameter>
    <parameter name="Original feature list">PROCESS IN PLACE</parameter>
</batchstep>
<batchstep method="io.github.mzmine.modules.dataprocessing.filter_isotopefinder.IsotopeFinderModule" parameter_version="1">
    <parameter name="Feature lists" type="BATCH_LAST_FEATURELISTS"/>
    <parameter name="Chemical elements">H,C,N,O,S,P</parameter>
    <parameter name="m/z tolerance (feature-to-scan)">
        <absolutetolerance>0.0</absolutetolerance>
        <ppmtolerance>6.0</ppmtolerance>
    </parameter>
    <parameter name="Maximum charge of isotope m/z">2</parameter>
    <parameter name="Search in scans">SINGLE MOST INTENSE</parameter>
</batchstep>
<batchstep method="io.github.mzmine.modules.dataprocessing.align_join.JoinAlignerModule" parameter_version="1">
    <parameter name="Feature lists" type="BATCH_LAST_FEATURELISTS"/>
    <parameter name="Feature list name">Aligned feature list</parameter>
    <parameter name="m/z tolerance (sample-to-sample)">
        <absolutetolerance>0.0</absolutetolerance>
        <ppmtolerance>6.0</ppmtolerance>
    </parameter>
    <parameter name="Weight for m/z">3.0</parameter>
    <parameter name="Retention time tolerance" unit="MINUTES">0.15</parameter>
    <parameter name="Weight for RT">1.0</parameter>
    <parameter name="Mobility tolerance" selected="false">1.0</parameter>
    <parameter name="Mobility weight">1.0</parameter>
    <parameter name="Require same charge state">false</parameter>
    <parameter name="Require same ID">false</parameter>
    <parameter name="Compare isotope pattern" selected="false">
        <parameter name="Isotope m/z tolerance">
            <absolutetolerance>0.0</absolutetolerance>
            <ppmtolerance>6.0</ppmtolerance>
        </parameter>
        <parameter name="Minimum absolute intensity"/>
        <parameter name="Minimum score"/>
    </parameter>
    <parameter name="Compare spectra similarity" selected="false">
        <parameter name="Spectral m/z tolerance">
            <absolutetolerance>0.0</absolutetolerance>
            <ppmtolerance>12.0</ppmtolerance>
        </parameter>
```

```
        <parameter name="MS level">2</parameter>
        <parameter name="Compare spectra similarity" selected_item="Weighted cosine similarity">
            <module name="Weighted cosine similarity">
                <parameter name="Weights">MassBank (mz^2 * I^0.5)</parameter>
                <parameter name="Minimum cos similarity">0.7</parameter>
                <parameter name="Handle unmatched signals">KEEP ALL AND MATCH TO ZERO</parameter>
            </module>
            <module name="Composite cosine identity (e.g., GC-EI-MS; similar to NIST search)">
                <parameter name="Weights">MassBank (mz^2 * I^0.5)</parameter>
                <parameter name="Minimum cos similarity">0.7</parameter>
                <parameter name="Handle unmatched signals">KEEP ALL AND MATCH TO ZERO</parameter>
            </module>
        </parameter>
    </parameter>
    <parameter name="Original feature list">REMOVE</parameter>
</batchstep>
<batchstep method="io.github.mzmine.modules.dataprocessing.filter_rowsfilter.RowsFilterModule" parameter_version="2">
    <parameter name="Feature lists" type="BATCH_LAST_FEATURELISTS"/>
    <parameter name="Name suffix">13C peak</parameter>
    <parameter name="Minimum aligned features (samples)" selected="false">
        <abs>1</abs>
        <rel>0.0</rel>
    </parameter>
    <parameter name="Minimum features in an isotope pattern" selected="false">2</parameter>
    <parameter name="Validate 13C isotope pattern" selected="true">
        <parameter name="m/z tolerance">
            <absolutetolerance>0.0</absolutetolerance>
            <ppmtolerance>4.0</ppmtolerance>
        </parameter>
        <parameter name="Max charge">2</parameter>
        <parameter name="Estimate minimum carbon">true</parameter>
        <parameter name="Remove if 13C">true</parameter>
        <parameter name="Exclude isotopes">O</parameter>
    </parameter>
    <parameter name="Remove redundant isotope rows">false</parameter>
    <parameter name="m/z" selected="false"/>
    <parameter name="Retention time" selected="false"/>
    <parameter name="features duration range" selected="false">
        <min>0.0</min>
        <max>3.0</max>
    </parameter>
    <parameter name="Chromatographic FWHM" selected="false">
        <min>0.0</min>
        <max>1.0</max>
    </parameter>
    <parameter name="Charge" selected="false">
        <min>1</min>
        <max>2</max>
    </parameter>
    <parameter name="Kendrick mass defect" selected="false">
        <parameter name="Kendrick mass defect">
            <min>0.0</min>
            <max>1.0</max>
        </parameter>
        <parameter name="Kendrick mass base"/>
        <parameter name="Shift">0.0</parameter>
        <parameter name="Charge">1</parameter>
        <parameter name="Divisor">1</parameter>
        <parameter name="Use Remainder of Kendrick mass">false</parameter>
    </parameter>
    <parameter name="Parameter">No parameters defined</parameter>
    <parameter name="Only identified?">false</parameter>
    <parameter name="Text in identity" selected="false"/>
    <parameter name="Text in comment" selected="false"/>
    <parameter name="Keep or remove rows">Keep rows that match all criteria</parameter>
    <parameter name="Feature with MS2 scan">true</parameter>
    <parameter name="Never remove feature with MS2">false</parameter>
    <parameter name="Reset the feature number ID">true</parameter>
    <parameter name="Mass defect" selected="false"/>
    <parameter name="Original feature list">PROCESS IN PLACE</parameter>
</batchstep>
<batchstep method="io.github.mzmine.modules.dataprocessing.gapfill_peakfinder.multithreaded.MultiThreadPeakFinderModule" parameter_version="1">
    <parameter name="Feature lists" type="BATCH_LAST_FEATURELISTS"/>
    <parameter name="Name suffix">gaps</parameter>
    <parameter name="Intensity tolerance">0.2</parameter>
    <parameter name="m/z tolerance (sample-to-sample)">
        <absolutetolerance>0.0</absolutetolerance>
        <ppmtolerance>12.0</ppmtolerance>
    </parameter>
    <parameter name="Retention time tolerance" unit="MINUTES">0.1</parameter>
    <parameter name="Minimum scans (data points)">1</parameter>
    <parameter name="Original feature list">PROCESS IN PLACE</parameter>
</batchstep>
<batchstep method="io.github.mzmine.modules.dataprocessing.filter_duplicatefilter.DuplicateFilterModule" parameter_version="1">
    <parameter name="Feature lists" type="BATCH_LAST_FEATURELISTS"/>
    <parameter name="Name suffix">dup</parameter>
    <parameter name="Filter mode">NEW AVERAGE</parameter>
    <parameter name="m/z tolerance">
        <absolutetolerance>0.0</absolutetolerance>
        <ppmtolerance>3</ppmtolerance>
    </parameter>
    <parameter name="RT tolerance" unit="MINUTES">0.035</parameter>
    <parameter name="Mobility tolerance" selected="true">0.008</parameter>
    <parameter name="Require same identification">false</parameter>
    <parameter name="Original feature list">PROCESS IN PLACE</parameter>
</batchstep>
```

```
<batchstep method="io.github.mzmine.modules.dataprocessing.group_metacorrelate.corrgrouping.CorrelateGroupingModule" parameter_version="2">
    <parameter name="Feature lists" type="BATCH_LAST_FEATURELISTS"/>
    <parameter name="RT tolerance" unit="MINUTES">0.075</parameter>
    <parameter name="Minimum feature height">10000.0</parameter>
    <parameter name="Intensity threshold for correlation">10000.0</parameter>
    <parameter name="Min samples filter">
        <parameter name="Min samples in all">
            <abs>1</abs>
            <rel>0.0</rel>
        </parameter>
        <parameter name="Min samples in group">
            <abs>0</abs>
            <rel>0.0</rel>
        </parameter>
        <parameter name="Min %-intensity overlap">0.5</parameter>
        <parameter name="Exclude gap-filled features">true</parameter>
    </parameter>
    <parameter name="Feature shape correlation" selected="true">
        <parameter name="Min data points">5</parameter>
        <parameter name="Min data points on edge">2</parameter>
        <parameter name="Measure">PEARSON</parameter>
        <parameter name="Min feature shape correlation">0.65</parameter>
        <parameter name="Min total correlation" selected="false">0.5</parameter>
    </parameter>
    <parameter name="Feature height correlation" selected="true">
        <parameter name="Minimum samples">2</parameter>
        <parameter name="Measure">PEARSON</parameter>
        <parameter name="Min correlation">0.65</parameter>
    </parameter>
    <parameter name="Suffix (or auto)" selected="true">metaCor</parameter>
</batchstep>
<batchstep method="io.github.mzmine.modules.dataprocessing.id_ion_identity_networking.ionidnetworking.IonNetworkingModule"
      parameter_version="1">
    <parameter name="Feature lists" type="BATCH_LAST_FEATURELISTS"/>
    <parameter name="m/z tolerance (intra-sample)">
        <absolutetolerance>0.0</absolutetolerance>
        <ppmtolerance>4.0</ppmtolerance>
    </parameter>
    <parameter name="Check">ALL FEATURES</parameter>
    <parameter name="Min height">50.0</parameter>
    <parameter name="Ion identity library">
        <parameter name="MS mode">NEGATIVE</parameter>
        <parameter name="Maximum charge">2</parameter>
        <parameter name="Maximum molecules/cluster">2</parameter>
        <parameter name="Adducts">
            <adduct_type selected="true">
                <subpart charge="-1" mass_difference="-1.007276" mol_formula="H" name="H" type="ADDUCT"/>
            </adduct_type>
            <adduct_type selected="true">
                <subpart charge="-1" mass_difference="-1.007276" mol_formula="H" name="H" type="ADDUCT"/>
                <subpart charge="-1" mass_difference="-1.007276" mol_formula="H" name="H" type="ADDUCT"/>
                <subpart charge="1" mass_difference="22.989218" mol_formula="Na" name="Na" type="ADDUCT"/>
            </adduct_type>
            <adduct_type selected="true">
                <subpart charge="1" mass_difference="22.989218" mol_formula="Na" name="Na" type="ADDUCT"/>
            </adduct_type>
            <adduct_type selected="true">
                <subpart charge="-1" mass_difference="34.969401" mol_formula="Cl" name="Cl" type="ADDUCT"/>
            </adduct_type>
            <adduct_type selected="true">
                <subpart charge="-1" mass_difference="78.918886" mol_formula="Br" name="Br" type="ADDUCT"/>
            </adduct_type>
            <adduct_type selected="true">
                <subpart charge="-1" mass_difference="44.99820285" mol_formula="CHO2" name="CHO2" type="ADDUCT"/>
            </adduct_type>
            <modification_type selected="true">
                <subpart charge="0" mass_difference="-15.023475" mol_formula="CH3" name="CH3" type="NEUTRAL_LOSS"/>
            </modification_type>
            <modification_type selected="true">
                <subpart charge="0" mass_difference="-17.026549" mol_formula="H3N" name="H3N" type="NEUTRAL_LOSS"/>
            </modification_type>
            <modification_type selected="true">
                <subpart charge="0" mass_difference="-18.010565" mol_formula="H2O" name="H2O" type="NEUTRAL_LOSS"/>
            </modification_type>
            <modification_type selected="true">
                <subpart charge="0" mass_difference="-18.010565" mol_formula="H2O" name="H2O" type="NEUTRAL_LOSS"/>
                <subpart charge="0" mass_difference="-18.010565" mol_formula="H2O" name="H2O" type="NEUTRAL_LOSS"/>
            </modification_type>
            <modification_type selected="true">
                <subpart charge="0" mass_difference="-146.05791" mol_formula="C6H10O4" name="C6H10O4" type="NEUTRAL_LOSS"/>
            </modification_type>
            <modification_type selected="true">
                <subpart charge="0" mass_difference="-162.052825" mol_formula="C6H10O5" name="C6H10O5" type="NEUTRAL_LOSS"/>
            </modification_type>
            <modification_type selected="true">
                <subpart charge="0" mass_difference="41.026549" mol_formula="C2H3N" name="C2H3N" type="CLUSTER"/>
            </modification_type>
            <modification_type selected="true">
                <subpart charge="0" mass_difference="46.005479" mol_formula="CH2O2" name="CH2O2" type="CLUSTER"/>
            </modification_type>
            <modification_type selected="true">
                <subpart charge="0" mass_difference="60.021129" mol_formula="C2H4O2" name="C2H4O2" type="CLUSTER"/>
            </modification_type>
        </parameter>
    </parameter>
    <parameter name="Annotation refinement" selected="true">
        <parameter name="Minimum size" selected="true">2</parameter>
        <parameter name="Delete small networks without major ion">true</parameter>
```

```
        <parameter name="Delete smaller networks: Link threshold" selected="true">3</parameter>
        <parameter name="Delete networks without monomer">true</parameter>
        <parameter name="Delete rows witout ion id">false</parameter>
    </parameter>
</batchstep>
<batchstep method="io.github.mzmine.modules.dataprocessing.id_ion_identity_networking.addionannotations.AddIonNetworkingModule"
       parameter_version="1">
    <parameter name="Feature lists" type="BATCH_LAST_FEATURELISTS"/>
    <parameter name="m/z tolerance (intra-sample)">
        <absolutetolerance>0.0</absolutetolerance>
        <ppmtolerance>12.0</ppmtolerance>
    </parameter>
    <parameter name="Min height">50.0</parameter>
    <parameter name="Annotation refinement" selected="true">
        <parameter name="Minimum size" selected="true">2</parameter>
        <parameter name="Delete small networks without major ion">true</parameter>
        <parameter name="Delete smaller networks: Link threshold" selected="true">3</parameter>
        <parameter name="Delete networks without monomer">true</parameter>
        <parameter name="Delete rows witout ion id">false</parameter>
    </parameter>
    <parameter name="Ion identity library">
        <parameter name="MS mode">NEGATIVE</parameter>
        <parameter name="Maximum charge">2</parameter>
        <parameter name="Maximum molecules/cluster">2</parameter>
        <parameter name="Adducts">
            <adduct_type selected="true">
                <subpart charge="-1" mass_difference="-1.007276" mol_formula="H" name="H" type="ADDUCT"/>
            </adduct_type>
            <adduct_type selected="true">
                <subpart charge="-1" mass_difference="-1.007276" mol_formula="H" name="H" type="ADDUCT"/>
                <subpart charge="-1" mass_difference="-1.007276" mol_formula="H" name="H" type="ADDUCT"/>
                <subpart charge="1" mass_difference="22.989218" mol_formula="Na" name="Na" type="ADDUCT"/>
            </adduct_type>
            <adduct_type selected="true">
                <subpart charge="1" mass_difference="22.989218" mol_formula="Na" name="Na" type="ADDUCT"/>
            </adduct_type>
            <adduct_type selected="true">
                <subpart charge="-1" mass_difference="34.969401" mol_formula="Cl" name="Cl" type="ADDUCT"/>
            </adduct_type>
            <adduct_type selected="true">
                <subpart charge="-1" mass_difference="78.918886" mol_formula="Br" name="Br" type="ADDUCT"/>
            </adduct_type>
            <adduct_type selected="true">
                <subpart charge="-1" mass_difference="44.99820285" mol_formula="CHO2" name="CHO2" type="ADDUCT"/>
            </adduct_type>
        </parameter>
    </parameter>
</batchstep>
<batchstep method="io.github.mzmine.modules.dataprocessing.id_ion_identity_networking.addionannotations.AddIonNetworkingModule"
       parameter_version="1">
    <parameter name="Feature lists" type="BATCH_LAST_FEATURELISTS"/>
    <parameter name="m/z tolerance (intra-sample)">
        <absolutetolerance>0.0</absolutetolerance>
        <ppmtolerance>12.0</ppmtolerance>
    </parameter>
    <parameter name="Min height">50.0</parameter>
    <parameter name="Annotation refinement" selected="true">
        <parameter name="Minimum size" selected="false">2</parameter>
        <parameter name="Delete small networks without major ion">true</parameter>
        <parameter name="Delete smaller networks: Link threshold" selected="true">3</parameter>
        <parameter name="Delete networks without monomer">true</parameter>
        <parameter name="Delete rows witout ion id">false</parameter>
    </parameter>
    <parameter name="Ion identity library">
        <parameter name="MS mode">NEGATIVE</parameter>
        <parameter name="Maximum charge">2</parameter>
        <parameter name="Maximum molecules/cluster">2</parameter>
        <parameter name="Adducts">
            <adduct_type selected="true">
                <subpart charge="-1" mass_difference="-1.007276" mol_formula="H" name="H" type="ADDUCT"/>
            </adduct_type>
            <adduct_type selected="true">
                <subpart charge="-1" mass_difference="-1.007276" mol_formula="H" name="H" type="ADDUCT"/>
                <subpart charge="-1" mass_difference="-1.007276" mol_formula="H" name="H" type="ADDUCT"/>
                <subpart charge="1" mass_difference="22.989218" mol_formula="Na" name="Na" type="ADDUCT"/>
            </adduct_type>
            <adduct_type selected="true">
                <subpart charge="1" mass_difference="22.989218" mol_formula="Na" name="Na" type="ADDUCT"/>
            </adduct_type>
            <adduct_type selected="true">
                <subpart charge="-1" mass_difference="34.969401" mol_formula="Cl" name="Cl" type="ADDUCT"/>
            </adduct_type>
            <adduct_type selected="true">
                <subpart charge="-1" mass_difference="78.918886" mol_formula="Br" name="Br" type="ADDUCT"/>
            </adduct_type>
            <adduct_type selected="true">
                <subpart charge="-1" mass_difference="44.99820285" mol_formula="CHO2" name="CHO2" type="ADDUCT"/>
            </adduct_type>
            <modification_type selected="true">
                <subpart charge="0" mass_difference="-15.0109" mol_formula="HN" name="NH" type="NEUTRAL_LOSS"/>
            </modification_type>
            <modification_type selected="true">
                <subpart charge="0" mass_difference="-15.023475" mol_formula="CH3" name="CH3" type="NEUTRAL_LOSS"/>
            </modification_type>
            <modification_type selected="true">
                <subpart charge="0" mass_difference="-17.026549" mol_formula="H3N" name="H3N" type="NEUTRAL_LOSS"/>
            </modification_type>
            <modification_type selected="true">
```

```
    <subpart charge="0" mass_difference="-18.010565" mol_formula="H2O" name="H2O" type="NEUTRAL_LOSS"/>
</modification_type>
<modification_type selected="true">
    <subpart charge="0" mass_difference="-18.010565" mol_formula="H2O" name="H2O" type="NEUTRAL_LOSS"/>
    <subpart charge="0" mass_difference="-18.010565" mol_formula="H2O" name="H2O" type="NEUTRAL_LOSS"/>
</modification_type>
<modification_type selected="true">
    <subpart charge="0" mass_difference="-18.010565" mol_formula="H2O" name="H2O" type="NEUTRAL_LOSS"/>
    <subpart charge="0" mass_difference="-18.010565" mol_formula="H2O" name="H2O" type="NEUTRAL_LOSS"/>
    <subpart charge="0" mass_difference="-18.010565" mol_formula="H2O" name="H2O" type="NEUTRAL_LOSS"/>
</modification_type>
<modification_type selected="true">
    <subpart charge="0" mass_difference="-18.010565" mol_formula="H2O" name="H2O" type="NEUTRAL_LOSS"/>
    <subpart charge="0" mass_difference="-18.010565" mol_formula="H2O" name="H2O" type="NEUTRAL_LOSS"/>
    <subpart charge="0" mass_difference="-18.010565" mol_formula="H2O" name="H2O" type="NEUTRAL_LOSS"/>
    <subpart charge="0" mass_difference="-18.010565" mol_formula="H2O" name="H2O" type="NEUTRAL_LOSS"/>
</modification_type>
<modification_type selected="true">
    <subpart charge="0" mass_difference="-18.010565" mol_formula="H2O" name="H2O" type="NEUTRAL_LOSS"/>
    <subpart charge="0" mass_difference="-18.010565" mol_formula="H2O" name="H2O" type="NEUTRAL_LOSS"/>
    <subpart charge="0" mass_difference="-18.010565" mol_formula="H2O" name="H2O" type="NEUTRAL_LOSS"/>
    <subpart charge="0" mass_difference="-18.010565" mol_formula="H2O" name="H2O" type="NEUTRAL_LOSS"/>
    <subpart charge="0" mass_difference="-18.010565" mol_formula="H2O" name="H2O" type="NEUTRAL_LOSS"/>
</modification_type>
<modification_type selected="true">
    <subpart charge="0" mass_difference="-27.0109" mol_formula="CHN" name="CHN" type="NEUTRAL_LOSS"/>
</modification_type>
<modification_type selected="true">
    <subpart charge="0" mass_difference="-27.994915" mol_formula="CO" name="CO" type="NEUTRAL_LOSS"/>
</modification_type>
<modification_type selected="true">
    <subpart charge="0" mass_difference="-27.994915" mol_formula="CO" name="CO" type="NEUTRAL_LOSS"/>
    <subpart charge="0" mass_difference="-27.994915" mol_formula="CO" name="CO" type="NEUTRAL_LOSS"/>
</modification_type>
<modification_type selected="true">
    <subpart charge="0" mass_difference="-27.994915" mol_formula="CO" name="CO" type="NEUTRAL_LOSS"/>
    <subpart charge="0" mass_difference="-27.994915" mol_formula="CO" name="CO" type="NEUTRAL_LOSS"/>
    <subpart charge="0" mass_difference="-27.994915" mol_formula="CO" name="CO" type="NEUTRAL_LOSS"/>
</modification_type>
<modification_type selected="true">
    <subpart charge="0" mass_difference="-14.01565" mol_formula="CH2" name="CH2" type="NEUTRAL_LOSS"/>
    <subpart charge="0" mass_difference="-14.01565" mol_formula="CH2" name="CH2" type="NEUTRAL_LOSS"/>
</modification_type>
<modification_type selected="true">
    <subpart charge="0" mass_difference="-14.01565" mol_formula="CH2" name="CH2" type="NEUTRAL_LOSS"/>
    <subpart charge="0" mass_difference="-15.02348" mol_formula="CH3" name="CH3" type="NEUTRAL_LOSS"/>
</modification_type>
<modification_type selected="true">
    <subpart charge="0" mass_difference="-30.01057" mol_formula="CH2O" name="CH2O" type="NEUTRAL_LOSS"/>
</modification_type>
<modification_type selected="true">
    <subpart charge="0" mass_difference="-32.02621" mol_formula="CH4O" name="CH4O" type="NEUTRAL_LOSS"/>
</modification_type>
<modification_type selected="true">
    <subpart charge="0" mass_difference="-42.010565" mol_formula="C2H2O" name="C2H2O" type="NEUTRAL_LOSS"/>
</modification_type>
<modification_type selected="true">
    <subpart charge="0" mass_difference="-43.00581" mol_formula="CHON" name="CHON" type="NEUTRAL_LOSS"/>
</modification_type>
<modification_type selected="true">
    <subpart charge="0" mass_difference="-43.989829" mol_formula="CO2" name="CO2" type="NEUTRAL_LOSS"/>
</modification_type>
<modification_type selected="true">
    <subpart charge="0" mass_difference="-43.989829" mol_formula="CO2" name="CO2" type="NEUTRAL_LOSS"/>
    <subpart charge="0" mass_difference="-43.989829" mol_formula="CO2" name="CO2" type="NEUTRAL_LOSS"/>
</modification_type>
<modification_type selected="true">
    <subpart charge="0" mass_difference="-27.994915" mol_formula="CO" name="CO" type="NEUTRAL_LOSS"/>
    <subpart charge="0" mass_difference="-18.010565" mol_formula="H2O" name="H2O" type="NEUTRAL_LOSS"/>
</modification_type>
<modification_type selected="true">
    <subpart charge="0" mass_difference="-46.00548" mol_formula="CH2O2" name="CH2O2" type="NEUTRAL_LOSS"/>
</modification_type>
<modification_type selected="true">
    <subpart charge="0" mass_difference="-46.04187" mol_formula="C2H6O" name="C2H6O" type="NEUTRAL_LOSS"/>
</modification_type>
<modification_type selected="true">
    <subpart charge="0" mass_difference="-60.02113" mol_formula="C2H4O2" name="C2H4O2" type="NEUTRAL_LOSS"/>
</modification_type>
<modification_type selected="true">
    <subpart charge="0" mass_difference="-66.0317" mol_formula="CH6O3" name="CH6O3" type="NEUTRAL_LOSS"/>
</modification_type>
<modification_type selected="true">
    <subpart charge="0" mass_difference="-30.01057" mol_formula="CH2O" name="CH2O" type="NEUTRAL_LOSS"/>
    <subpart charge="0" mass_difference="-43.989829" mol_formula="CO2" name="CO2" type="NEUTRAL_LOSS"/>
</modification_type>
<modification_type selected="true">
    <subpart charge="0" mass_difference="-14.01565" mol_formula="CH2" name="CH2" type="NEUTRAL_LOSS"/>
    <subpart charge="0" mass_difference="-15.02348" mol_formula="CH3" name="CH3" type="NEUTRAL_LOSS"/>
    <subpart charge="0" mass_difference="-46.00548" mol_formula="CH2O2" name="CH2O2" type="NEUTRAL_LOSS"/>
</modification_type>
<modification_type selected="true">
    <subpart charge="0" mass_difference="-87.06841" mol_formula="C4H9NO" name="C4H9NO" type="NEUTRAL_LOSS"/>
</modification_type>
<modification_type selected="true">
    <subpart charge="0" mass_difference="-88.01604" mol_formula="C3H4O3" name="C3H4O3" type="NEUTRAL_LOSS"/>
</modification_type>
<modification_type selected="true">
```

```
    <subpart charge="0" mass_difference="-88.05243" mol_formula="C4H8O2" name="C4H8O2" type="NEUTRAL_LOSS"/>
</modification_type>
<modification_type selected="true">
    <subpart charge="0" mass_difference="-89.04768" mol_formula="C3H7NO2" name="C3H7NO2" type="NEUTRAL_LOSS"/>
</modification_type>
<modification_type selected="true">
    <subpart charge="0" mass_difference="-90.031695" mol_formula="C3H6O3" name="C3H6O3" type="NEUTRAL_LOSS"/>
</modification_type>
<modification_type selected="true">
    <subpart charge="0" mass_difference="-97.96738" mol_formula="H2O4S" name="H2O4S" type="NEUTRAL_LOSS"/>
</modification_type>
<modification_type selected="true">
    <subpart charge="0" mass_difference="-97.9769" mol_formula="H3O4P" name="H3O4P" type="NEUTRAL_LOSS"/>
</modification_type>
<modification_type selected="true">
    <subpart charge="0" mass_difference="-102.06808" mol_formula="C5H10O2" name="C5H10O2" type="NEUTRAL_LOSS"/>
</modification_type>
<modification_type selected="true">
    <subpart charge="0" mass_difference="-104.01096" mol_formula="C3H4O4" name="C3H4O4" type="NEUTRAL_LOSS"/>
</modification_type>
<modification_type selected="true">
    <subpart charge="0" mass_difference="-43.989829" mol_formula="CO2" name="CO2" type="NEUTRAL_LOSS"/>
    <subpart charge="0" mass_difference="-43.989829" mol_formula="CO2" name="CO2" type="NEUTRAL_LOSS"/>
    <subpart charge="0" mass_difference="-18.010565" mol_formula="H2O" name="H2O" type="NEUTRAL_LOSS"/>
</modification_type>
<modification_type selected="true">
    <subpart charge="0" mass_difference="-108.04226" mol_formula="C3H8O4" name="C3H8O4" type="NEUTRAL_LOSS"/>
</modification_type>
<modification_type selected="true">
    <subpart charge="0" mass_difference="-115.06333" mol_formula="C5H9NO2" name="C5H9NO2" type="NEUTRAL_LOSS"/>
</modification_type>
<modification_type selected="true">
    <subpart charge="0" mass_difference="-15.023475" mol_formula="CH3" name="CH3" type="NEUTRAL_LOSS"/>
    <subpart charge="0" mass_difference="-15.023475" mol_formula="CH3" name="CH3" type="NEUTRAL_LOSS"/>
    <subpart charge="0" mass_difference="-43.989829" mol_formula="CO2" name="CO2" type="NEUTRAL_LOSS"/>
    <subpart charge="0" mass_difference="-43.989829" mol_formula="CO2" name="CO2" type="NEUTRAL_LOSS"/>
</modification_type>
<modification_type selected="true">
    <subpart charge="0" mass_difference="-120.04226" mol_formula="C4H8O4" name="C4H8O4" type="NEUTRAL_LOSS"/>
</modification_type>
<modification_type selected="true">
    <subpart charge="0" mass_difference="-43.989829" mol_formula="CO2" name="CO2" type="NEUTRAL_LOSS"/>
    <subpart charge="0" mass_difference="-43.989829" mol_formula="CO2" name="CO2" type="NEUTRAL_LOSS"/>
    <subpart charge="0" mass_difference="-18.010565" mol_formula="H2O" name="H2O" type="NEUTRAL_LOSS"/>
    <subpart charge="0" mass_difference="-18.010565" mol_formula="H2O" name="H2O" type="NEUTRAL_LOSS"/>
</modification_type>
<modification_type selected="true">
    <subpart charge="0" mass_difference="-126.03169" mol_formula="C6H6O3" name="C6H6O3" type="NEUTRAL_LOSS"/>
</modification_type>
<modification_type selected="true">
    <subpart charge="0" mass_difference="-132.04226" mol_formula="C5H8O4" name="C5H8O4" type="NEUTRAL_LOSS"/>
</modification_type>
<modification_type selected="true">
    <subpart charge="0" mass_difference="-136.05243" mol_formula="C8H8O2" name="C8H8O2" type="NEUTRAL_LOSS"/>
</modification_type>
<modification_type selected="true">
    <subpart charge="0" mass_difference="-144.04226" mol_formula="C6H8O4" name="C6H8O4" type="NEUTRAL_LOSS"/>
</modification_type>
<modification_type selected="true">
    <subpart charge="0" mass_difference="-146.03678" mol_formula="C9H6O2" name="C9H6O2" type="NEUTRAL_LOSS"/>
</modification_type>
<modification_type selected="true">
    <subpart charge="0" mass_difference="-146.05791" mol_formula="C6H10O4" name="C6H10O4" type="NEUTRAL_LOSS"/>
</modification_type>
<modification_type selected="true">
    <subpart charge="0" mass_difference="-146.05791" mol_formula="C6H10O4" name="C6H10O4" type="NEUTRAL_LOSS"/>
    <subpart charge="0" mass_difference="-146.05791" mol_formula="C6H10O4" name="C6H10O4" type="NEUTRAL_LOSS"/>
</modification_type>
<modification_type selected="true">
    <subpart charge="0" mass_difference="-148.05243" mol_formula="C9H8O2" name="C9H8O2" type="NEUTRAL_LOSS"/>
</modification_type>
<modification_type selected="true">
    <subpart charge="0" mass_difference="-152.01096" mol_formula="C7H4O4" name="C7H4O4" type="NEUTRAL_LOSS"/>
</modification_type>
<modification_type selected="true">
    <subpart charge="0" mass_difference="-152.04734" mol_formula="C8H8O3" name="C8H8O3" type="NEUTRAL_LOSS"/>
</modification_type>
<modification_type selected="true">
    <subpart charge="0" mass_difference="-162.03169" mol_formula="C9H6O3" name="C9H6O3" type="NEUTRAL_LOSS"/>
</modification_type>
<modification_type selected="true">
    <subpart charge="0" mass_difference="-162.05282" mol_formula="C6H10O5" name="C6H10O5" type="NEUTRAL_LOSS"/>
</modification_type>
<modification_type selected="true">
    <subpart charge="0" mass_difference="-162.05282" mol_formula="C6H10O5" name="C6H10O5" type="NEUTRAL_LOSS"/>
    <subpart charge="0" mass_difference="-162.05282" mol_formula="C6H10O5" name="C6H10O5" type="NEUTRAL_LOSS"/>
</modification_type>
<modification_type selected="true">
    <subpart charge="0" mass_difference="-162.05282" mol_formula="C6H10O5" name="C6H10O5" type="NEUTRAL_LOSS"/>
    <subpart charge="0" mass_difference="-162.05282" mol_formula="C6H10O5" name="C6H10O5" type="NEUTRAL_LOSS"/>
    <subpart charge="0" mass_difference="-162.05282" mol_formula="C6H10O5" name="C6H10O5" type="NEUTRAL_LOSS"/>
</modification_type>
<modification_type selected="true">
    <subpart charge="0" mass_difference="-168.04226" mol_formula="C8H8O4" name="C8H8O4" type="NEUTRAL_LOSS"/>
</modification_type>
<modification_type selected="true">
    <subpart charge="0" mass_difference="-170.05791" mol_formula="C8H10O4" name="C8H10O4" type="NEUTRAL_LOSS"/>
</modification_type>
<modification_type selected="true">
```

```
            <subpart charge="0" mass_difference="-176.03209" mol_formula="C6H8O6" name="C6H8O6" type="NEUTRAL_LOSS"/>
        </modification_type>
        <modification_type selected="true">
            <subpart charge="0" mass_difference="-176.04734" mol_formula="C10H8O3" name="C10H8O3" type="NEUTRAL_LOSS"/>
        </modification_type>
        <modification_type selected="true">
            <subpart charge="0" mass_difference="-179.07937" mol_formula="C6H13NO5" name="C6H13NO5" type="NEUTRAL_LOSS"/>
        </modification_type>
        <modification_type selected="true">
            <subpart charge="0" mass_difference="-180.06339" mol_formula="C6H12O6" name="C6H12O6" type="NEUTRAL_LOSS"/>
        </modification_type>
        <modification_type selected="true">
            <subpart charge="0" mass_difference="-192.06339" mol_formula="C7H12O6" name="C7H12O6" type="NEUTRAL_LOSS"/>
        </modification_type>
        <modification_type selected="true">
            <subpart charge="0" mass_difference="-197.08994" mol_formula="C6H12O6" name="C6H12O6" type="NEUTRAL_LOSS"/>
        </modification_type>
        <modification_type selected="true">
            <subpart charge="0" mass_difference="-198.07395" mol_formula="C6H14O7" name="C6H14O7" type="NEUTRAL_LOSS"/>
        </modification_type>
        <modification_type selected="true">
            <subpart charge="0" mass_difference="-204.06339" mol_formula="C8H12O6" name="C8H12O6" type="NEUTRAL_LOSS"/>
        </modification_type>
        <modification_type selected="true">
            <subpart charge="0" mass_difference="-206.05791" mol_formula="C11H10O4" name="C11H10O4" type="NEUTRAL_LOSS"/>
        </modification_type>
        <modification_type selected="true">
            <subpart charge="0" mass_difference="-238.22967" mol_formula="C16H30O" name="C16H30O" type="NEUTRAL_LOSS"/>
        </modification_type>
        <modification_type selected="true">
            <subpart charge="0" mass_difference="-248.05322" mol_formula="C9H12O8" name="C9H12O8" type="NEUTRAL_LOSS"/>
        </modification_type>
        <modification_type selected="true">
            <subpart charge="0" mass_difference="-266.07904" mol_formula="C13H14O6" name="C13H14O6" type="NEUTRAL_LOSS"/>
        </modification_type>
        <modification_type selected="true">
            <subpart charge="0" mass_difference="-308.0896" mol_formula="C15H16O7" name="C15H16O7" type="NEUTRAL_LOSS"/>
        </modification_type>
        <modification_type selected="true">
            <subpart charge="0" mass_difference="-314.06378" mol_formula="C13H14O9" name="C13H14O9" type="NEUTRAL_LOSS"/>
        </modification_type>
        <modification_type selected="true">
            <subpart charge="0" mass_difference="-324.08452" mol_formula="C15H16O8" name="C15H16O8" type="NEUTRAL_LOSS"/>
        </modification_type>
        <modification_type selected="true">
            <subpart charge="0" mass_difference="-338.10017" mol_formula="C16H18O8" name="C16H18O8" type="NEUTRAL_LOSS"/>
        </modification_type>
        <modification_type selected="true">
            <subpart charge="0" mass_difference="-368.11073" mol_formula="C17H20O9" name="C17H20O9" type="NEUTRAL_LOSS"/>
        </modification_type>
        <modification_type selected="true">
            <subpart charge="0" mass_difference="15.99491462" mol_formula="O" name="O" type="CLUSTER"/>
        </modification_type>
        <modification_type selected="true">
            <subpart charge="0" mass_difference="45.05785" mol_formula="C2H7N" name="C2H7N" type="CLUSTER"/>
        </modification_type>
        <modification_type selected="true">
            <subpart charge="0" mass_difference="46.005479" mol_formula="CH2O2" name="CH2O2" type="CLUSTER"/>
        </modification_type>
        <modification_type selected="true">
            <subpart charge="0" mass_difference="60.021129" mol_formula="C2H4O2" name="C2H4O2" type="CLUSTER"/>
        </modification_type>
        <modification_type selected="true">
            <subpart charge="0" mass_difference="32.026215" mol_formula="CH4O" name="CH4O" type="CLUSTER"/>
        </modification_type>
        <modification_type selected="true">
            <subpart charge="0" mass_difference="41.026549" mol_formula="C2H3N" name="C2H3N" type="CLUSTER"/>
        </modification_type>
        <modification_type selected="true">
            <subpart charge="0" mass_difference="60.058064" mol_formula="C3H8O" name="C3H8O" type="CLUSTER"/>
        </modification_type>
    </parameter>
</parameter>
</batchstep>
<batchstep method="io.github.mzmine.modules.dataprocessing.filter_rowsfilter.RowsFilterModule" parameter_version="2">
    <parameter name="Feature lists" type="BATCH_LAST_FEATURELISTS"/>
    <parameter name="Name suffix">filtered</parameter>
    <parameter name="Minimum aligned features (samples)" selected="false">
        <abs>1</abs>
        <rel>0.0</rel>
    </parameter>
    <parameter name="Minimum features in an isotope pattern" selected="false">2</parameter>
    <parameter name="Validate 13C isotope pattern" selected="false">
        <parameter name="m/z tolerance">
            <absolutetolerance>0.0</absolutetolerance>
            <ppmtolerance>12.0</ppmtolerance>
        </parameter>
        <parameter name="Max charge">1</parameter>
        <parameter name="Estimate minimum carbon">true</parameter>
        <parameter name="Remove if 13C">true</parameter>
        <parameter name="Exclude isotopes">H,C,N,O,S,P</parameter>
    </parameter>
    <parameter name="Remove redundant isotope rows">false</parameter>
    <parameter name="m/z" selected="false"/>
    <parameter name="Retention time" selected="false"/>
    <parameter name="features duration range" selected="false">
        <min>0.0</min>
        <max>3.0</max>
```

```
        </parameter>
        <parameter name="Chromatographic FWHM" selected="false">
            <min>0.0</min>
            <max>1.0</max>
        </parameter>
        <parameter name="Charge" selected="false">
            <min>1</min>
            <max>2</max>
        </parameter>
        <parameter name="Kendrick mass defect" selected="false">
            <parameter name="Kendrick mass defect">
                <min>0.0</min>
                <max>1.0</max>
            </parameter>
            <parameter name="Kendrick mass base"/>
            <parameter name="Shift">0.0</parameter>
            <parameter name="Charge">1</parameter>
            <parameter name="Divisor">1</parameter>
            <parameter name="Use Remainder of Kendrick mass">false</parameter>
        </parameter>
        <parameter name="Parameter">No parameters defined</parameter>
        <parameter name="Only identified?">false</parameter>
        <parameter name="Text in identity" selected="false"/>
        <parameter name="Text in comment" selected="false"/>
        <parameter name="Keep or remove rows">Keep rows that match all criteria</parameter>
        <parameter name="Feature with MS2 scan">true</parameter>
        <parameter name="Never remove feature with MS2">true</parameter>
        <parameter name="Reset the feature number ID">true</parameter>
        <parameter name="Mass defect" selected="false"/>
        <parameter name="Original feature list">PROCESS IN PLACE</parameter>
    </batchstep>
    <batchstep method="io.github.mzmine.modules.io.export_features_gnps.fbmn.GnpsFbmnExportAndSubmitModule" parameter_version="2">
        <parameter name="Feature lists" type="BATCH_LAST_FEATURELISTS"/>
        <parameter name="Filename">
            <current_file>data/interim/mzmine/lists/MYFILENAME_gnps</current_file>
        </parameter>
        <parameter name="Merge MS/MS (experimental)" selected="true">
            <parameter name="Select spectra to merge">across samples</parameter>
            <parameter name="m/z merge mode">weighted average (remove outliers)</parameter>
            <parameter name="intensity merge mode">sum intensities</parameter>
            <parameter name="Expected mass deviation">
                <absolutetolerance>0.0</absolutetolerance>
                <ppmtolerance>6.0</ppmtolerance>
            </parameter>
            <parameter name="Cosine threshold (%)">0.7</parameter>
            <parameter name="Signal count threshold (%)">0.2</parameter>
            <parameter name="Isolation window offset (m/z)">0.0</parameter>
            <parameter name="Isolation window width (m/z)">3.0</parameter>
        </parameter>
        <parameter name="Filter rows">MS2 OR ION IDENTITY</parameter>
        <parameter name="Feature intensity">Area</parameter>
        <parameter name="CSV export">ALL</parameter>
        <parameter name="Submit to GNPS" selected="false">
            <parameter name="Meta data file" selected="false"/>
            <parameter name="Export ion identity networks">true</parameter>
            <parameter name="Presets">HIGHRES</parameter>
            <parameter name="Job title"/>
            <parameter name="Email"/>
            <parameter name="Username"/>
            <parameter name="Password"/>
            <parameter name="Open website">true</parameter>
        </parameter>
        <parameter name="Open folder">false</parameter>
    </batchstep>
    <batchstep method="io.github.mzmine.modules.io.export_features_sirius.SiriusExportModule" parameter_version="1">
        <parameter name="Feature lists" type="BATCH_LAST_FEATURELISTS"/>
        <parameter name="Filename">
            <current_file>data/interim/mzmine/mgfs/MYFILENAME_sirius.mgf</current_file>
        </parameter>
        <parameter name="Merge MS/MS" selected="true">
            <parameter name="Select spectra to merge">across samples</parameter>
            <parameter name="m/z merge mode">weighted average (remove outliers)</parameter>
            <parameter name="intensity merge mode">sum intensities</parameter>
            <parameter name="Expected mass deviation">
                <absolutetolerance>0.0</absolutetolerance>
                <ppmtolerance>6.0</ppmtolerance>
            </parameter>
            <parameter name="Cosine threshold (%)">0.7</parameter>
            <parameter name="Signal count threshold (%)">0.2</parameter>
            <parameter name="Isolation window offset (m/z)">0.0</parameter>
            <parameter name="Isolation window width (m/z)">3.0</parameter>
        </parameter>
        <parameter name="m/z tolerance">
            <absolutetolerance>0.0</absolutetolerance>
            <ppmtolerance>6.0</ppmtolerance>
        </parameter>
        <parameter name="Only rows with annotation">false</parameter>
        <parameter name="Exclude multiple charge">false</parameter>
        <parameter name="Exclude multimers">false</parameter>
    </batchstep>
</batch>
```

3.3 MZmine launch command

```bash
#!/usr/bin/env bash
# -*- coding: utf-8 -*-
# A simple script to create custom mzmine batch files from template
# (use of gsed because of MacOS)

FILEPATH=$1
MODE=$2
FILENAME=$(basename "$1" .mzML)
OUTPATH=$"params/params/batch_${FILENAME}_mzmine.xml"
echo $FILEPATH &&
echo $FILENAME &&
echo $OUTPATH &&
cp params/default/batch_template_mzmine_$MODE.xml $OUTPATH &&
gsed -i "s+MYFILEPATH+$FILEPATH+g" $OUTPATH &&
gsed -i "s+MYFILENAME+$FILENAME+g" $OUTPATH &&
echo "Done"
```

Supporting Information 4. Sirius commands used (positive and negative)

4.1 Sirius template (positive)

```bash
#!/usr/bin/env bash
# -*- coding: utf-8 -*-

INPUT=$1
sirius \
--input $1 \
--output ${INPUT%.*} \
config \
--IsotopeSettings.filter true \
--FormulaSearchDB BIO,COCONUT \
--Timeout.secondsPerTree 0 \
--FormulaSettings.enforced HCNOPS \
--Timeout.secondsPerInstance 0 \
--AdductSettings.detectable "[M + H]+, [M + H4N]+, [M + Na]+, [M + K]+, [M - H + Na2]+, [M - H + K2]+, [M - CH3 + H]+, [M - H2O + H]+, [M - H4O2 + H]+, [M - H6O3 + H]+, [M - H8O4 + H]+, [M - H10O5 + H]+, [M - CO + H]+, [M - C2O2 + H]+, [M - C3O3 + H]+, [M - C2H4 + H]+, [M - C2H5 + H]+, [M - CH2O + H]+, [M - CH4O + H]+, [M - C2H2O + H]+, [M - CO2 + H]+, [M - C2O4 + H]+, [M - CH2O2 + H]+, [M - C2H6O + H]+, [M - C2H4O2 + H]+, [M - CH6O3 + H]+, [M - C2H2O3 + H]+, [M - C3H7O2 + H]+, [M - C3H4O3 + H]+, [M - C4H8O2 + H]+, [M - H2O4S + H]+, [M - H3O4P + H]+, [M - C5H10O2 + H]+, [M - C3H4O4 + H]+, [M - C5H8O4 + H]+, [M - C9H6O2 + H]+, [M - C6H10O4 + H]+, [M - C12H20O8 + H]+, [M - C9H8O2 + H]+, [M - C7H4O4 + H]+, [M - C9H6O3 + H]+, [M - C6H10O5 + H]+, [M - C12H20O10 + H]+, [M - C18H30O15 + H]+" \
--UseHeuristic.mzToUseHeuristicOnly 650 \
--AlgorithmProfile orbitrap \
--IsotopeMs2Settings IGNORE \
--MS2MassDeviation.allowedMassDeviation 5.0ppm \
--NumberOfCandidatesPerIon 1 \
--UseHeuristic.mzToUseHeuristic 300 \
--FormulaSettings.detectable B,Br,Cl,F,I,Se,Si \
--NumberOfCandidates 10 \
--ZodiacNumberOfConsideredCandidatesAt300Mz 10 \
--ZodiacRunInTwoSteps true \
--ZodiacEdgeFilterThresholds.minLocalConnections 10 \
--ZodiacEdgeFilterThresholds.thresholdFilter 0.95 \
--ZodiacEpochs.burnInPeriod 2000 \
--ZodiacEpochs.numberOfMarkovChains 10 \
--ZodiacNumberOfConsideredCandidatesAt800Mz 50 \
--ZodiacEpochs.iterations 20000 \
--AdductSettings.enforced , \
--AdductSettings.fallback "[M + H]+, [M + Na]+, [M + K]+" \
--FormulaResultThreshold true \
--InjectElGordoCompounds true \
--StructureSearchDB BIO,COCONUT \
--RecomputeResults false \
formula \
zodiac \
fingerprint \
structure \
canopus \
W
```

4.2 Sirius template (negative)

```
#!/usr/bin/env bash
# -*- coding: utf-8 -*-

INPUT=$1
sirius \
--input $1 \
--output ${INPUT%.*} \
config \
--IsotopeSettings.filter true \
--FormulaSearchDB BIO,COCONUT \
--Timeout.secondsPerTree 0 \
--FormulaSettings.enforced HCNOPS \
--Timeout.secondsPerInstance 0 \
--AdductSettings.detectable "[M]-, [M + Cl]-, [M + Br]-, [M + CH2O2 - H]-, [M - H2 +
    Na]-, [M + C2H4O2 - H]-, [M + C2HF3O2 - H]-, [M + C2H3N - H]-, [M - 2H + K ]-, [M -
    H2O - H]-, [M - H]-, [M - H4O2 - H]-, [M - H6O3 - H]-, [M - H8O4 - H]-, [M - H10O5 -
    H]-, [M - CO - H]-, [M - C2O2 - H]-, [M - C3O3 - H]-, [M - C2H4 - H]-, [M - C2H5 -
    H]-, [M - CH2O - H]-, [M - CH4O - H]-, [M - C2H2O - H]-, [M - CO2 - H]-, [M - C2O4 -
    H]-, [M - CH2O2 - H]-, [M - C2H6O - H]-, [M - C2H4O2 - H]-, [M - CH6O3 - H]-, [M -
    C2H2O3 - H]-, [M - C3H7O2 - H]-, [M - C3H4O3 - H]-, [M - C4H8O2 - H]-, [M - H2O4S -
    H]-, [M - H3O4P - H]-, [M - C5H10O2 - H]-, [M - C3H4O4 - H]-, [M - C5H8O4 - H]-, [M
    - C9H6O2 - H]-, [M - C6H10O4 - H]-, [M - C12H20O8 - H]-, [M - C9H8O2 - H]-, [M -
    C7H4O4 - H]-, [M - C9H6O3 - H]-, [M - C6H10O5 - H]-, [M - C12H20O10 - H]-, [M -
    C18H30O15 - H]-" \
--UseHeuristic.mzToUseHeuristicOnly 650 \
--AlgorithmProfile orbitrap \
--IsotopeMs2Settings IGNORE \
--MS2MassDeviation.allowedMassDeviation 5.0ppm \
--NumberOfCandidatesPerIon 1 \
--UseHeuristic.mzToUseHeuristic 300 \
--FormulaSettings.detectable B,Br,Cl,F,I,Se,Si \
--NumberOfCandidates 10 \
--ZodiacNumberOfConsideredCandidatesAt300Mz 10 \
--ZodiacRunInTwoSteps true \
--ZodiacEdgeFilterThresholds.minLocalConnections 10 \
--ZodiacEdgeFilterThresholds.thresholdFilter 0.95 \
--ZodiacEpochs.burnInPeriod 2000 \
--ZodiacEpochs.numberOfMarkovChains 10 \
--ZodiacNumberOfConsideredCandidatesAt800Mz 50 \
--ZodiacEpochs.iterations 20000 \
--AdductSettings.enforced , \
--AdductSettings.fallback "[M]-, [M - H2 + Na]-, [M + C2H4O2 - H]-, [M + C2HF3O2 - H]-,
    [M - H2O - H]-, [M - H]-" \
--FormulaResultThreshold true \
--InjectElGordoCompounds true \
--StructureSearchDB BIO,COCONUT \
--RecomputeResults false \
formula \
zodiac \
fingerprint \
structure \
canopus \
W
```

References

(1) Bass, E. ethanbass/chromatographR: v0.4.7. 2023; https://doi.org/10.5281/zenodo.7734158, accessed 2023-04-13.

(2) Melnikov, A. D.; Tsentalovich, Y. P.; Yanshole, V. V. Deep Learning for the Precise Peak Detection in High-Resolution LC–MS Data. *Analytical Chemistry* **2019**, *92*, 588–592.

(3) Gloaguen, Y.; Kirwan, J. A.; Beule, D. Deep Learning-Assisted Peak Curation for Large-Scale LC-MS Metabolomics. *Analytical Chemistry* **2022**, *94*, 4930–4937.
